# Disruption of ATRX-RNA interactions uncovers roles in ATRX localization and PRC2 function

**DOI:** 10.1038/s41467-020-15902-9

**Published:** 2020-05-06

**Authors:** Wenqing Ren, Nicole Medeiros, Robert Warneford-Thomson, Phillip Wulfridge, Qingqing Yan, Joyce Bian, Simone Sidoli, Benjamin A. Garcia, Emmanuel Skordalakes, Eric Joyce, Roberto Bonasio, Kavitha Sarma

**Affiliations:** 1 Gene expression and Regulation program, The Wistar Institute, Philadelphia, PA 19104 USA; 2 Epigenetics Institute, University of Pennsylvania, Philadelphia, PA 19104 USA; 3 Department of Cell and Developmental Biology, University of Pennsylvania, Philadelphia, PA 19104 USA; 4 Graduate group in Biochemistry and Molecular Biophysics, University of Pennsylvania, Philadelphia, PA 19104 USA; 5 University of Pennsylvania, Philadelphia, PA 19104 USA; 6 Department of Genetics, University of Pennsylvania, Philadelphia, PA 19104 USA; 7 Present Address: Department of Biochemistry, Albert Einstein College of Medicine, Bronx, NY 10461 USA

**Keywords:** Biochemistry, RNA, Gene silencing

## Abstract

Heterochromatin in the eukaryotic genome is rigorously controlled by the concerted action of protein factors and RNAs. Here, we investigate the RNA binding function of ATRX, a chromatin remodeler with roles in silencing of repetitive regions of the genome and in recruitment of the polycomb repressive complex 2 (PRC2). We identify ATRX RNA binding regions (RBRs) and discover that the major ATRX RBR lies within the N-terminal region of the protein, distinct from its PHD and helicase domains. Deletion of this ATRX RBR (ATRXΔRBR) compromises ATRX interactions with RNAs in vitro and in vivo and alters its chromatin binding properties. Genome-wide studies reveal that loss of RNA interactions results in a redistribution of ATRX on chromatin. Finally, our studies identify a role for ATRX-RNA interactions in regulating PRC2 localization to a subset of polycomb target genes.

## Abstract (web summary)

ATRX is an RNA binding protein that mediates targeting of polycomb repressive complex 2 (PRC2) to genomic sites. Here the authors identify the RNA binding region and show that the RNA binding is required for ATRX localization and for its recruitment of PRC2 to a subset of polycomb targets.

## Introduction

Dynamic interactions between epigenetic regulators and RNAs are important to establish and maintain correct gene expression programs during development^1–3^. Many developmental disorders and other diseases, including cancers, occur due to a failure to safeguard these regulatory programs. ATRX (alpha-thalassemia X-linked mental retardation) is a chromatin remodeler whose loss during early development is associated with a severe developmental disorder called ATRX Syndrome^4^. Recently, studies have shown that ATRX is frequently mutated in cancers and its loss is intimately linked to the alternative lengthening of telomeres (ALT), a mechanism that contributes to cellular immortality in cancer^5–9^. Indeed, ATRX loss is associated with an increase in aneuploidy and cancer aggressiveness^10^.

ATRX is an RNA-binding protein but the functional role of its interaction with RNA is unknown. ATRX has roles in the process of X chromosome inactivation (XCI)^11^, a developmental process where one of the two X chromosomes in female mammals is silenced to balance gene dosage between the sexes^12–14^. This process is regulated by a large number of non-coding RNAs and their interactions with specific proteins^15^. The Xist long non-coding RNA (lncRNA) that is required for initiation of XCI, coats the inactive X chromosome (Xi) in female mammals and serves as a platform for the recruitment for various silencing proteins including the polycomb repressive complex 2 (PRC2)^12,16^. The PRC2 complex contains the EZH2 histone methyltransferase that catalyzes trimethylation of histone H3 at lysine 27 (H3K27me3) and is required for the maintenance of correct gene expression patterns during development^17^. It is also mutated or upregulated in a large number of cancers, which makes it an attractive target for cancer therapeutics^18,19^. The PRC2 complex is enriched on the Xi and, similar to ATRX, interacts with Xist lncRNA^20–22^. Interestingly, while ATRX itself is not enriched across the Xi, depletion of ATRX results in the loss of PRC2 from genes on the Xi as well as from other polycomb target genes on autosomes^11^. Thus, ATRX functions in targeting PRC2 to specific genomic sites. The mechanisms by which ATRX regulates PRC2 and the role of ATRX–RNA interactions in this process is unclear.

ATRX is also enriched at tandem repetitive regions of the genome^23^. Transcription from tandem repeats can form regulatory chromatin structures known as G quadruplexes (G4)^24^. Loss of ATRX is associated with elevated G4 DNA containing DNA–RNA hybrid chromatin structures called R loops at telomeres^23,25,26^. Transcription of telomeric repeats results in the production of a non-coding RNA, TERRA, and in the formation of R loops at these regions^27,28^. ATRX binds telomeric R loops and this interaction is abolished when R loops are destroyed. This suggests that ATRX targeting to repetitive regions can occur through its interaction with G4 DNA, DNA–RNA hybrids, or both. ATRX also functions at centromeric and pericentromeric regions that are highly repetitive, typically depleted for genes and until recently thought to be largely refractory to transcription. However, several reports indicate that these regions produce small amounts of RNAs of variable length in both the G1/S and M phases of the cell cycle^29–31^. Satellite transcripts originating from these regions must be strictly controlled since accumulation of these repetitive RNAs is seen in many cancers and is associated with centromere instability^32–34^. ATRX is enriched at these constitutive heterochromatin regions and contributes to their silencing. Loss of ATRX increases satellite RNA transcription and promotes genome instability^35–37^.

Studying the function of protein–RNA interactions has been challenging since many RNA-binding proteins contain non-canonical RNA-binding regions (RBRs) that cannot be predicted by sequence alone^38,39^. Many studies have investigated RNA-mediated regulation of chromatin complexes by perturbing individual interacting RNA^11,40–42^. However, these complexes often bind to hundreds if not thousands of RNAs, which makes it difficult to obtain a general view for the functions of these interactions. Recent advances that allow proteome-wide unbiased identification of RBRs, have made it possible to overcome this challenge^38,39,43,44^. Here, we use the RBR-ID method^39^ to identify the RNA-binding region (RBR) of ATRX and investigate the significance of ATRX–RNA interactions in its localization to specific regions of the genome and in the recruitment of PRC2.

## Results

### ATRX chromatin association is regulated by RNA interactions

**Fig. 1:**
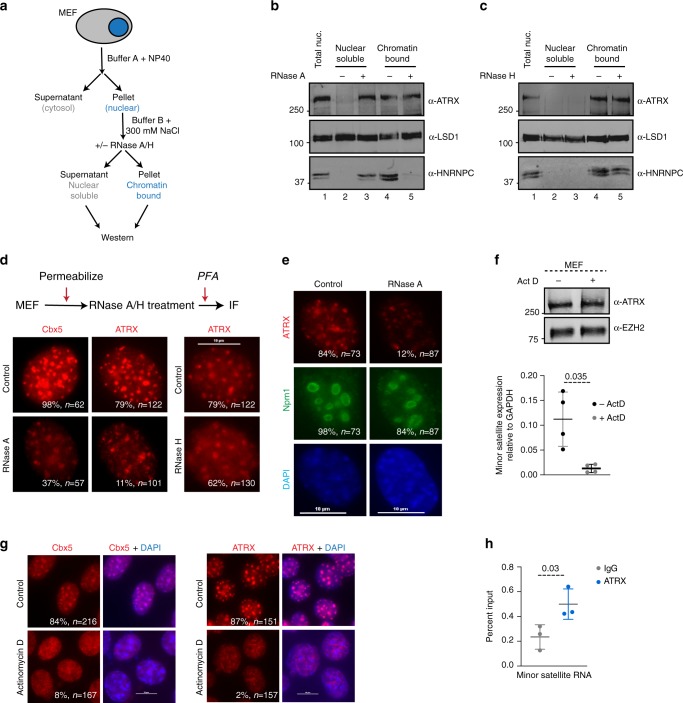
RNA interactions regulate ATRX chromatin association. **a** Schematic for nuclear fractionation of MEFs with and without RNase A or RNase H treatment. **b** Western blot for ATRX, LSD1, and HNRNPC in MEF nuclear soluble and chromatin-bound fractions obtained with and without RNase A treatment as indicated on top. Representative blot from four independent experiments is shown. **c** Western blot for ATRX, LSD1, and HNRNPC in MEF nuclear soluble and chromatin-bound fractions obtained with and without RNase H treatment as indicated. Representative blot from three independent experiments is shown. **d** Top—Schematic of RNase A or RNase H treatment of MEFs for immunostaining. Bottom—Immunostaining of control (top) and RNase A or RNase H treated (bottom) MEFs with ATRX (red) and Cbx5 (red) antibodies. Percent of nuclei with pericentromeric Cbx5 or ATRX signal is shown along with total number of nuclei (*n*) quantified. Scale bar = 10 μm. **e** Immunostaining of control (left) and RNase A treated (right) MEFs with ATRX (red) and Npm1 (green) antibodies. Nucleus is stained with DAPI. Percent of nuclei with pericentromeric ATRX signal or nucleolar Npm1 signal is shown along with total number of nuclei (*n*) quantified. Scale bar = 10 μm. **f** Top—Western blot for ATRX and EZH2 in WT MEFs with and without Actinomycin D treatment. Bottom—RT-PCR analysis of minor satellite transcripts in WT MEFs with and without Actinomycin D treatment. Data are presented as mean values +/− SEM. *P* values are calculated using two-sided Student’s *t* test. **g** Immunostaining of WT MEFs with ATRX and Cbx5 antibodies (red) before and after Actinomycin D treatment. Percent of nuclei with pericentromeric Cbx5 or ATRX signal is shown along with total number of nuclei (*n*) quantified. Scale bar = 10 μm. **h** Cross-linking and immunoprecipitation in WT MEFs with IgG (gray) or ATRX antibodies (dark blue). qPCR analysis with minor satellite primers. Data are presented as mean values +/− SEM. *P* values are calculated using two-sided Student’s *t* test. Source data underlying Fig. 1b–h are provided as a Source Data file.

The chromatin remodeler ATRX interacts with RNAs in both differentiated and undifferentiated cell types^11,39,41^. To uncover the roles of ATRX–RNA interactions in its function, we first determined if ATRX nuclear distribution was affected by disruption of RNAs. For this, we examined the nuclear distribution of ATRX upon treatment with RNase A. We fractionated mouse embryonic fibroblasts (MEFs) into cytosolic, nuclear extract (soluble), and nuclear pellet (chromatin-bound) fractions (Fig. 1a). In our experimental setup, subsequent to cytosolic extraction, we treated permeabilized nuclei with RNase A. The protein fraction that is soluble at 300 mM salt concentration was collected and the proteins that remained bound to the nuclear pellet were recovered with high salt and sonication. We used the well characterized RNA-binding protein HNRNPC, that is known to interact with chromatin through RNA interactions, as a control^45^. We found that without RNase A treatment, HNRNPC remained in the chromatin bound fraction (Fig. 1b, compare lanes 2 and 4). Upon treatment with RNase A, most HNRNPC was released from chromatin into the soluble fraction (Fig. 1b, compare lanes 3 and 5). Next, we analyzed ATRX distribution in these same extracts using an antibody generated against a fragment in the C terminus of ATRX (Supplementary Fig. 1a). We observed that at 300 mM salt without RNase A treatment, the majority of ATRX remained chromatin bound (Fig. 1b, compare lanes 2 and 4). Intriguingly, under the same salt conditions but with RNase A treatment, there is an increase in ATRX protein levels in the soluble fraction (Fig. 1b, compare lanes 2 and 3). The distribution of LSD1, an unrelated histone demethylase, did not change upon RNase A treatment. Since ATRX has known associations with R loops, chromatin structures that contain a DNA:RNA hybrid, we tested whether its localization to chromatin is disrupted upon treatment with RNase H, an enzyme that specifically degrades RNA within a DNA:RNA hybrid. We used the fractionation scheme as in Fig. 1a and treated nuclei with RNase H. We found that upon RNase H treatment, HNRNPC remained chromatin bound (Fig. 1c, compare lanes 3 and 5). Similarly, ATRX is not released into the soluble fraction and remains bound to chromatin (Fig. 1c, compare lanes 3 and 5). Based on this we conclude that a population of ATRX may associate with chromatin through RNA interactions. However, the species of RNA that tethers ATRX to chromatin is not predominantly in the form of DNA:RNA hybrids.

To visualize changes in ATRX distribution at the cellular level, we examined its localization in MEFs by immunofluorescence microscopy. ATRX is enriched at DAPI dense regions of the nucleus that corresponds to centromeric and pericentromeric heterochromatin^46^ (Supplementary Fig. 1b). To test how RNase A and RNase H treatments affects ATRX enrichment at these regions, we treated permeabilized MEFs with RNase A or RNase H prior to paraformaldehyde fixation (Fig. 1d). In previous studies, RNase A treatment of nuclei resulted in loss of Cbx5 enrichment at pericentric heterochromatin suggesting that Cbx5 localization to these sites is dependent on an RNA component^47^. In agreement with these studies we observe that while Cbx5 is localized to pericentric heterochromatin in control nuclei, upon RNase A treatment Cbx5 enrichment at these regions is markedly reduced (Fig. 1d, left compare top and bottom panels). Similarly, we found that in untreated MEFs, ATRX shows clear enrichment at pericentric heterochromatin (Fig. 1d, middle). Upon RNase A treatment, we observed that ATRX localization at pericentromeres becomes weaker (Fig. 1d, middle, compare top and bottom panels). We also found that treatment with RNase H resulted in a reduction of ATRX signal at pericentromeres (Fig. 1d, right), albeit lower than RNase A treatment, consistent with the observation that RNase H treatment of nuclei does not release a significant amount of ATRX from chromatin. To ascertain whether the effects of RNase A treatment on ATRX localization was specific, we analyzed the distribution of the nucleolar phosphoprotein nucleophosmin (Npm1) under the same conditions. Npm1 localizes to nucleoli through G quadruplex DNA binding at rDNA loci^48^. We co-stained for ATRX and Npm1 in control and RNase A treated nuclei. We found that the pattern of Npm1 nucleolar staining remains unchanged upon RNase A treatment (Fig. 1e). In the same nuclei, the localization of ATRX to pericentromeres is reduced. Our results support a role of RNAs in regulating ATRX localization to specific nuclear structures including pericentromeres. ATRX is known to localize to PML bodies^49^. We observed that after RNase A treatment of nuclei, some ATRX puncta remained. To test if these correspond to PML bodies, we co-stained nuclei for ATRX and PML proteins before and after RNase A treatment (Supplementary Fig. 1c). Some but not all residual ATRX foci colocalize to PML bodies, which could be a result of incomplete digestion of RNAs or due to ATRX presence in another nuclear sub-compartment.

Pericentromeres have previously been described to contain a non-coding RNA component^47^. To determine whether transcription of repetitive pericentromeric RNAs plays a role in the recruitment of ATRX to these regions, we decreased levels of RNAs by treating MEFs with Actinomycin D. We normalized levels of minor satellite RNA to GAPDH RNA that is known to have a long half-life and whose levels are not changed after 6 h of Actinomycin D treatment. We found that 6 h of Actinomycin D treatment resulted in >5-fold decrease in minor satellite RNA levels (Fig. 1f, bottom). At this same time point, ATRX protein levels were not changed (Fig. 1f, top). To determine whether decrease in centromeric transcription alters ATRX localization to these heterochromatic regions, we visualized ATRX enrichment by immunofluorescence microscopy. As shown previously, in WT MEFs, both ATRX and Cbx5 are enriched at DAPI dense pericentric heterochromatin. Upon treatment with Actinomycin D, enrichment of both proteins at centromeres is lost, consistent with a role for transcription in the recruitment and stabilization of ATRX and Cbx5 to constitutive centromeric heterochromatin (Fig. 1g). Since our results indicate that an RNA component is required for ATRX localization to these constitutive heterochromatin regions, we tested whether ATRX is able to interact with RNAs that originate from these regions. Using cross-linking and immunoprecipitation (CLIP) followed by qPCR, we tested the direct interaction between ATRX and minor satellite RNAs (Fig. 1h). Our results show that ATRX interacts with minor satellite RNAs, providing support to our assertion that ATRX localizes to constitutive heterochromatin through RNA interactions. The low binding levels may be attributed to low levels of expression of satellite RNAs combined with the inefficiency of UV cross-linking in vivo.

### ATRX binds RNA through distinct N- and C-terminal domains

To investigate the mechanism and function of ATRX–RNA interactions, we first sought to identify sites of RNA binding within this protein. Recently, two techniques have been developed to identify RBRs within proteins lacking canonical RNA-binding domains, RBDmap and RBR-ID, both of which utilize proteins–RNA cross-linking in vivo followed by mass spectrometry (MS) to identify the site of protein–RNA interactions^38,39^. We chose to reanalyze the publicly available RBR-ID dataset to assess possible RNA interaction sites on ATRX^39^. For this experiment, mouse embryonic stem cells (mESCs) are grown in the presence of 4-thiouridine (4SU), a photoactivatable uridine analog that is specifically incorporated into RNAs^50^. Exposure to long wavelength UV (312 nm) results in the cross-linking of proteins that directly contact 4SU-containing RNAs. The nuclear proteome is processed for MS and RNA-bound peptides are identified by comparing with mass spectra obtained from cells not pulsed with 4SU. Peptides that are specifically depleted in the cross-linked samples map to potential RBRs within proteins. We re-analyzed the in vivo RBR-ID data obtained from mESCs nuclei^39^ and found several ATRX peptides that were significantly depleted due to RNA cross-linking (Supplementary Fig. 2a). Therefore, in vivo RBR-ID identified ATRX peptides that have the potential to interact with RNAs.

Proteome-wide RBR-ID is a powerful tool to discover RNA-binding proteins and their RBRs. However, the power of this approach to clearly define all possible RBRs in any given protein is limited by its relative abundance in the context of the entire proteome^22^. To increase our ability to detect all possible RBRs within ATRX, we developed an in vitro RBR-ID approach. Previous RNA electromobility shift assay (EMSA) studies have shown that full-length human ATRX (hATRX) protein binds the Repeat A region of the Xist lncRNA, a 435 nt repetitive motif at the 5ʹ end, with high affinity (Kd ~ 5 nM)^11^. We expressed and purified full-length human FLAG-ATRX-HA protein (F-ATRX-HA) from *S. frugiperda* (Sf9) insect cells. Purified hATRX was incubated with a 4SU-labeled fragment of Xist Repeat A RNA. We used a 192-nucleotide fragment that was sufficient to bind ATRX with high affinity (Kd ~ 17.90 nM) (Supplementary Fig. 2b, red box)^11^. ATRX-4SU Repeat A RNA-binding reactions were cross-linked by exposure to 312 nm UV and processed for mass spectrometry. Eight independent biological replicates across two experiments were used in the analysis to identify ATRX RBRs with high confidence.

**Fig. 2:**
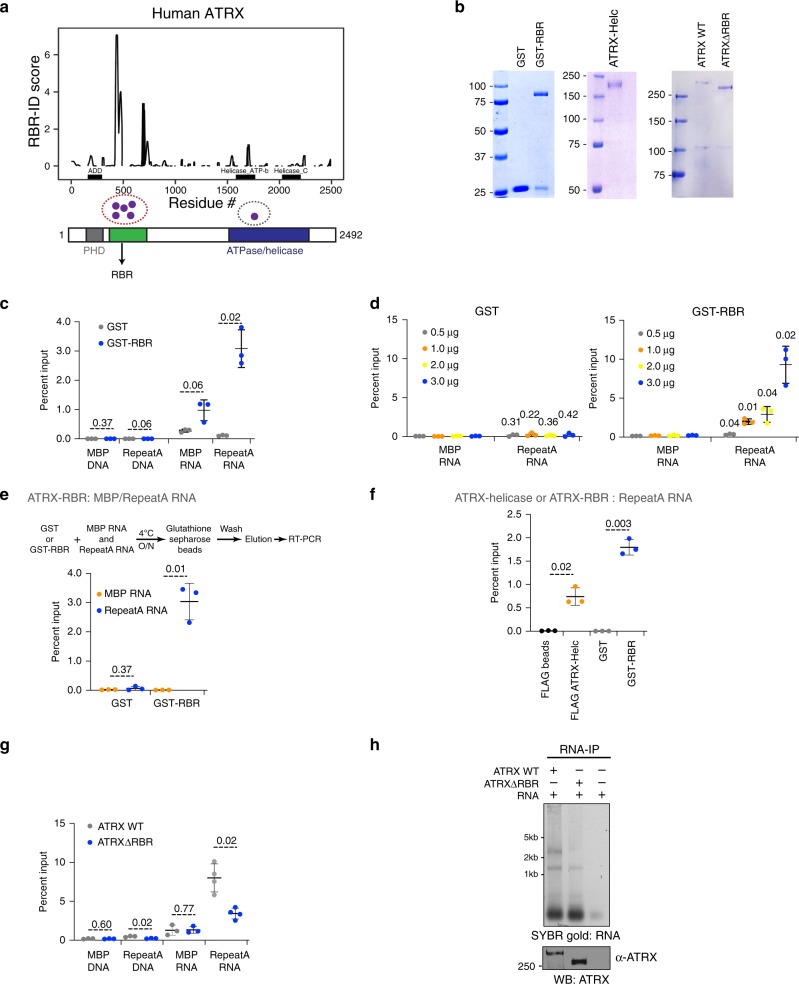
RBR-ID identifies regions within ATRX that bind RNA. **a** Top—Residue-level RBR-ID scores plotted along the primary sequence of human ATRX. Data are from eight biological replicates processed in two separate experiments. Bottom—Schematic for location of RNA binding peptides along human ATRX. PHD finger domain is in gray and helicase domain in dark blue. The region we designate the RBR is in green. **b** Coomassie gel of purified GST and GST-RBR (left), ATRX helicase domain (middle), and full-length ATRX and ATRXΔRBR (right) proteins. Representative image from three independent protein purifications is shown. **c** In vitro DNA/RNA IP with GST (gray) and GST-RBR (blue) proteins as indicated with MBP or Xist Repeat A DNA/RNA. Data are presented as mean values +/− SEM. *P* values are calculated using two-sided Student’s *t* test. **d** In vitro RNA IP with GST (left) and GST-RBR (right) proteins at indicated concentrations with MBP or Xist Repeat A RNA. Data are presented as mean values +/− SEM. *P* values are calculated using two-sided Student’s *t* test. **e** In vitro RNA IP with GST and GST-RBR and a mixture of MBP and Repeat A RNAs. Data are presented as mean values +/− SEM. *P* values are calculated using two-sided Student’s *t* test. **f** In vitro RNA IP with FLAG-ATRX helicase, GST-RBR and Repeat A RNA. Flag beads alone or GST are used as controls. Data are presented as mean values +/− SEM. *P* values are calculated using two-sided Student’s *t* test. **g** In vitro DNA/RNA IP with ATRX (gray) and ATRXΔRBR (blue) proteins as indicated with MBP or Xist Repeat A DNA/RNA. Data are presented as mean values +/− SEM. *P* values are calculated using two-sided Student’s *t* test. **h** RNA IP with purified proteins as indicated and total RNAs from MEFs. RNA is visualized by SYBR gold staining and immunoprecipitated proteins are detected by western blot. Source data are provided as a Source Data file. Representative image from three independent experiments is shown. Source data underlying Fig. 2b–h are provided as a Source Data file.

This in vitro RBR-ID experiment identified six significantly (*P* < 0.1) depleted peptides (Fig. 2a). Of these, one mapped to the C terminus and the majority (5 out of 6) of the identified peptides mapped to an N-terminal region adjacent to the PHD finger domain (residues 431–739) (Fig. 2a, Supplementary Fig. 2c). In addition to identifying specific peptides that pass predetermined thresholds, RBR-ID allows for calculation of a numeric score for each protein residue by combining extent and reproducibility of depletion across all peptides that overlap that residue^39^, which can provide additional information regarding the position of RBRs within protein that display weak or distributed binding to RNA. We plotted the RBR-ID score along the primary sequence of ATRX and found multiple potential regions of interaction both in the N terminus and in the C terminus, including one that overlapped the helicase domain (Fig. 2a), a region previously shown to bind RNA^11^. Based on the number of recovered peptides at the N terminus and on the fact that in vivo RBR-ID also detected sub-threshold cross-linking signal in this region, we designated aa 400–750 of hATRX as its putative major RBR (ATRX–RBR) and sought to determine its biochemical and biological function.

### ATRX–RBR is necessary and sufficient for Xist RNA binding

To ascertain whether the RBR alone is able to interact with nucleic acids, we expressed and purified GST-tagged ATRX–RBR (Fig. 2b, left) and tested its ability to bind the 435nt fragment of Xist Repeat A DNA or RNA by in vitro pulldowns (Fig. 2c). As controls we used 300nt RNA and DNA fragments derived from the N terminus of the Maltose Binding Protein (MBP). We found that both GST and GST-RBR did not bind MBP or Repeat A DNAs (Fig. 2c, left). GST protein alone does not show appreciable binding to either Repeat A or MBP RNA. GST-RBR binds Repeat A RNA and to a significantly lower extent the MBP RNA (Fig. 2c, right). Thus, our results indicate that the RNA-binding region alone is sufficient to interact with RNA. To test the specificity of interaction of GST-RBR with Repeat A RNA, we used increasing concentrations (0.5–3 μg) of GST or GST-RBR protein with equal amounts (5 μg) of MBP or Xist RNA. We found that even at the lowest protein concentration (0.5 μg), GST-RBR bound Repeat A RNA more than MBP RNA (Fig. 2d). GST protein alone did not show binding to Repeat A RNA even at the highest concentration (3 μg). We tested whether GST-RBR specifically binds Repeat A RNA even in the presence MBP RNA in the same reaction. We combined 5 μg each of Repeat A RNA and MBP RNA and incubated these with either GST alone or GST-RBR. Our results show that GST-RBR preferentially interacts with Repeat A RNA and does not bind MBP RNA (Fig. 2e). We conclude that the RBR shows specificity for the physiological ATRX–RNA ligand Xist compared with a nonspecific control (MBP).

The helicase domain containing fragment of ATRX (F6) was previously shown to bind Repeat A RNA^11^. We purified the F6 fragment from Sf9 cells (Fig. 2b, middle) and compared the RNA-binding ability of the RBR that we identified in this study to the C-terminus helicase domain of ATRX. We found that while both GST-RBR and F6 bind Repeat A RNA, GST-RBR is able to pull down Repeat A RNA more efficiently (Fig. 2f). Next, we assessed the contribution of ATRX–RBR to its RNA-binding ability. We reasoned that since this region showed the highest interaction with Xist Repeat A RNA fragment by in vitro RBR-ID, its deletion could destabilize and perhaps abolish most ATRX–RNA interactions. We generated baculovirus expressing FLAG-hATRX-HA (ATRX) and a deletion mutant lacking amino acids 400–750 within full-length hATRX (ATRXΔRBR) and purified the tagged proteins by FLAG affinity chromatography from Sf9 cells (Fig. 2b, right). We performed in vitro DNA or RNA IP with these proteins and Xist Repeat A or MBP DNA or RNA. We found that both full-length ATRX and ATRXΔRBR showed weak binding to MBP or Xist Repeat A DNA (Fig. 2g). We also found that similar to the RBR alone, full-length ATRX bound robustly to Xist Repeat A compared with weak binding to MBP RNA (Fig. 2g). ATRXΔRBR showed a significantly weaker interaction with Xist Repeat A and did not interact with MBP RNA. The residual binding that we observe between ATRXΔRBR and Repeat A RNA could be mediated through the helicase domain (Fig. 2f). To determine if deletion of the RBR compromised general RNA binding function of ATRX, we performed a pulldown with recombinant ATRX or ATRXΔRBR proteins and total RNA isolated from MEFs (Fig. 2h and Supplementary Fig. 2d). Our results indicate that when equal amounts of ATRX and ATRXΔRBR are immunoprecipitated (Fig. 2h, western), ATRX pulls down significantly more RNAs than ATRXΔRBR (Fig. 2h, SYBR gold). We conclude that deletion of amino acids 400–750 in hATRX results in overall reduction of interactions between the protein and RNAs.

### ATRXΔRBR displays reduced RNA interactions in vivo

**Fig. 3:**
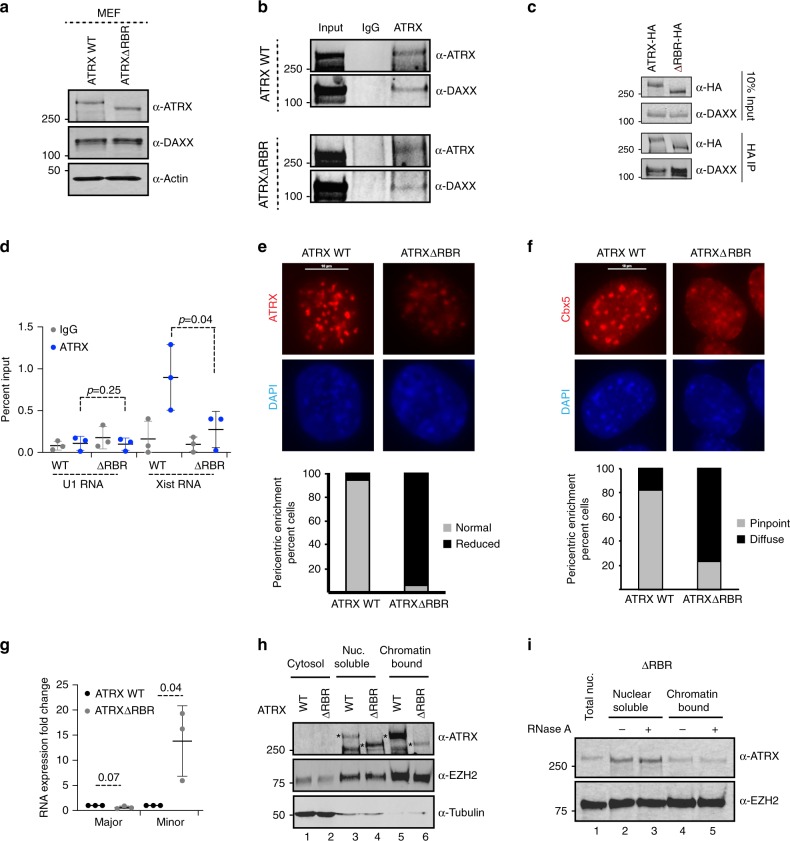
ATRXΔRBR has reduced RNA and chromatin interactions in vivo. **a** Western blot for ATRX, DAXX, and Actin in equal amount (50 μg) of WT and ATRXΔRBR nuclear extract. Representative image from four independent experiments is shown. **b** Endogenous immunoprecipitation (IP) of ATRX from WT and ATRXΔRBR MEFs. IgG is used as a control. Immunoprecipitates are analyzed with ATRX and DAXX antibodies. Source data are provided as a Source Data file. Representative image from three independent experiments is shown. **c** Immunoprecipitation (IP) with HA agarose from HA-ATRX and HA-ATRXΔRBR transfected HEK293. Immunoprecipitated proteins are analyzed with HA and DAXX antibodies. Representative image from three independent experiments is shown. **d** UV-RIP in WT and ATRXΔRBR MEFs as indicated with IgG (gray) or ATRX antibodies (dark blue). qPCR analysis with primers spanning Xist exon 1–3 and U1 snRNAs. Data are presented as mean values +/− SEM. *P* values are calculated using two-sided Student’s *t* test. **e** Top—Immunostaining of WT and ATRXΔRBR MEFs with ATRX antibodies (red) and DAPI (blue). Bottom—Quantification of nuclei that show normal (gray) or reduced (black) enrichment of ATRX on centromeric/pericentromeric heterochromatin. WT *n* = 127 and ATRXΔRBR *n* = 189. Scale bar = 10 μm. **f** Top—Immunostaining of Cbx5 (red) in WT and ATRXΔRBR MEFs. Nucleus is stained with DAPI (blue). Bottom—Quantification of nuclei that show normal (gray) or diffuse (black) enrichment of CBX5 on centromeric/pericentromeric heterochromatin. WT *n* = 151 and ATRXΔRBR *n* = 136. Scale bar = 10 μm. **g** RT-PCR analysis of major and minor satellite transcripts in WT and ATRXΔRBR. Data are presented as mean values +/− SEM. *P* values are calculated using two-sided Student’s *t* test. **h** Western blot for ATRX, EZH2, and Tubulin in cytosol, nuclear soluble, and chromatin-bound fractions from WT and ATRXΔRBR MEFs. Positions of full length and ATRXΔRBR are shown with asterisks (*). Representative image from three independent experiments is shown. **i** Western blot for ATRX, and EZH2 in ATRXΔRBR MEF nuclear soluble and chromatin bound fractions obtained with and without RNase A treatment as indicated on top. Representative image from three independent experiments is shown. Source data underlying Fig. 3a–i are provided as a Source Data file.

Deletion of the ATRX–RBR in vitro resulted in reduced ATRX–RNA interactions (Figs. 2g and h). We wanted to test whether we could compromise ATRX–RNA interactions in vivo if this same region was disrupted. We turned to a CRISPR Cas9 approach to delete the ATRX–RBR in vivo (Supplementary Fig. 3a)^51,52^. The ATRX–RBR lies within exon 9 of the mouse ATRX gene. To build on our in vitro findings on ATRX-Xist RNA interactions, we targeted female MEFs that express Xist RNA. We designed two guide RNAs (gRNAs) flanking the RBR corresponding to amino acids 400–712 in full-length mouse ATRX. We isolated one clone that contained an in-frame deletion that resulted in the production of a protein that migrated at ≈250 kDa (compared with 280 kDa of WT ATRX) (Fig. 3a). This fragment corresponds to a shortened ATRX protein due to the internal deletion, since no signal was detected in a knock out (KO) clone using the same antibody. We further characterized this clone that we named ATRXΔRBR. Sanger sequencing revealed that the ATRXΔRBR clone does not contain the region between amino acids 459 and 748 (Supplementary Fig. 3b). Our analysis also showed that by western blot, both WT and ATRXΔRBR clones expressed a comparable amount of ATRX protein (Fig. 3a). These results suggest that deletion of the RBR does not cause ATRX protein instability in vivo. ATRX is reported to interact with the histone chaperone DAXX^53–57^. The ATRX–DAXX complex is responsible for the deposition of the histone variant H3.3 at heterochromatic repetitive regions of the genome such as telomeres and pericentromeres and at endogenous retroviral elements and function in repression of these genomic regions^58^. We observed that DAXX protein stability is dependent on ATRX levels. In ATRX knockdown (KD) MEF cell lines where ATRX is acutely depleted, DAXX protein levels were also decreased (Supplementary Fig. 3c). However, DAXX levels were unchanged between WT and ATRXΔRBR (Fig. 3a). To test if ATRX and DAXX proteins interact in vivo in the absence of the RBR, we performed endogenous pulldowns using ATRX antibodies. We found that both ATRX and ATRXΔRBR pulled down DAXX protein (Fig. 3b, Supplementary Fig. 3d). To further confirm the interaction between ATRXΔRBR and DAXX protein, we expressed HA-tagged full-length ATRX or ATRXΔRBR in HEK293 and perform an HA tag immunoprecipitation to test whether DAXX protein was pulled down (Fig. 3c). We found that both WT full-length ATRX and ATRXΔRBR are able to bind DAXX protein in vivo. This is consistent with previous reports that DAXX interacts with ATRX through a C-terminus region close to the helicase domain^56^. We conclude that deletion of RBR does not compromise ATRX interactions with DAXX in vivo.

Deleting the ATRX–RBR reduces its ability to bind Xist Repeat A RNA in vitro (Fig. 2g). ATRX binds Xist and other RNAs in vivo^5,11,39^. Our ATRXΔRBR clone is a female MEF cell line and therefore expresses Xist lncRNA. This allowed us to investigate whether deletion of the RBR in endogenous ATRX affected interaction with Xist RNA in vivo. Using UV cross-linking and immunoprecipitation, we tested the direct interaction between ATRX and Xist RNAs in WT and ATRXΔRBR MEFs^59^ (Fig. 3d). Consistent with previous studies, WT ATRX interacted with Xist RNA in female MEFs. Interestingly, under the same experimental conditions, the ATRXΔRBR interaction with Xist RNA was significantly reduced (Fig. 3d). Neither ATRX nor ATRXΔRBR pulled down any appreciable amount of the abundant nuclear U1 RNA. These results demonstrate that ATRXΔRBR has significantly reduced Xist RNA-binding potential in vivo. The residual binding observed in ATRXΔRBR could be attributed to interactions between the helicase domain and Xist RNA (Fig. 2f).

### ATRXΔRBR shows reduced chromatin association

In MEFs, ATRX is clearly enriched and visualized as bright signals at DAPI dense regions that correspond to constitutive heterochromatin. We examined localization of ATRX to these regions in WT and ATRXΔRBR MEFs by immunofluorescence microscopy. While WT ATRX is able to localize to heterochromatic foci that we defined as regions of the nuclei with dense DAPI signals, ATRXΔRBR enrichment at these regions is significantly lower with over 90% of nuclei showing lower ATRX in ATRXΔRBR cells as compared with WT MEFs (Fig. 3e). Therefore, we conclude that RNA interactions guide ATRX to heterochromatic regions. We examined whether decreased ATRX enrichment from pericentromeres had an effect on Cbx5 localization at these sites. Overall, Cbx5 enrichment at pericentromeres was not affected (Fig. 3f). In WT MEFs, Cbx5 appeared as bright pinpoints that overlapped with DAPI dense regions that correspond to pericentromeres. However, we observed that the Cbx5 immunofluorescence signal, while still present, appeared more spread out and diffuse at pericentromeres in ATRXΔRBR as compared with WT MEFs (Fig. 3f, bottom), suggesting that ATRX presence may help to accumulate or stabilize Cbx5. We also note that the HP1 binding motif (PxVxL) is contained within the RBR^60^. Therefore, we cannot exclude the possibility that at pericentromeres, ATRX enrichment may be determined by a combination of RNA and HP1 interactions.

Pericentric and centromeric heterochromatin are comprised of major and minor satellite repeats, respectively. In recent years, several studies have shown that satellite heterochromatin, that was previously thought to be transcriptionally inert, is transcribed at low levels in many cell types and highly transcribed in some cancers^30,31,34^. This low level of transcription is important for localization of several silencing and chromosome segregation complexes to these regions^61^. We analyzed the expression of satellite RNAs from WT and ATRXΔRBR MEFs. We found that while there was not a significant effect on major satellite transcription, minor satellite transcripts were upregulated over 10-fold in ATRXΔRBR (Fig. 3g). Therefore, ATRX recruitment to centromeres and pericentromeres through its RNA interactions plays an important role in maintenance of repression at these regions.

Our previous results indicate that a substantial fraction of ATRX in the nucleus is tethered to chromatin through its RNA interactions, as evidenced by its release from chromatin upon RNase A treatment (Fig. 1b). Therefore, we hypothesize that an ATRX protein that lacks or is diminished in its ability to bind RNA (ATRXΔRBR) should become untethered from chromatin and become visible in the nuclear soluble fraction. We fractionated WT and ATRXΔRBR MEFs into cytosolic, nuclear soluble and chromatin bound fractions. Deletion of the RBR did not disrupt nuclear localization of ATRX protein (Fig. 3h), further confirming that ATRX∆RBR is a functional protein except for its inability to bind RNA. We found that under our experimental conditions, the majority of WT ATRX is present in the chromatin-bound fraction (Fig. 3h, lane 5). However, under the same conditions, ATRXΔRBR is distributed between nuclear soluble and chromatin-bound fractions, mimicking the behavior of WT ATRX in response to RNase A treatment of nuclei (Fig. 3h, compare lanes 4 and 6). Quantification of ATRX distribution further confirmed that in WT MEFs, ATRX is predominantly nuclear and chromatin bound. In contrast, ATRXΔRBR, although still nuclear, is significantly enriched in the nuclear soluble fraction (Supplementary Fig. 3e). In fact, treatment with RNase A was ineffectual in releasing the residual chromatin-bound fraction of ATRX∆RBR (Fig. 3i), suggesting that (1) all RNA-mediated interactions that tether WT ATRX to chromatin require the RBR at residues 400–750, and (2) a fraction of ATRX remains associated to chromatin in absence of RNA interactions, implying that recruitment of ATRX to genomic targets occurs by at least two distinct modes, one is RNA-dependent and the other is RNA-independent.

### Disruption of RNA interactions with ATRX causes its redistribution on chromatin

**Fig. 4:**
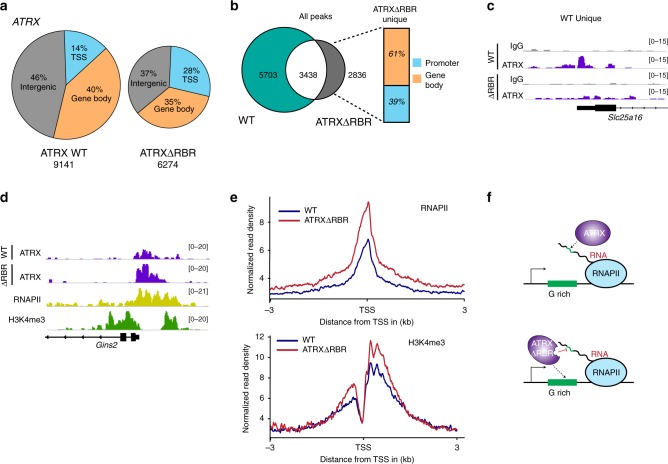
ATRX genome-wide distribution relies on RNA-dependent and independent mechanisms. **a** ATRX and ATRXΔRBR peak distribution in MEFs at TSS, gene body and intergenic sites. **b** Venn diagram of peak overlap between ATRX and ATRXΔRBR. Total number of peaks in each sample and their overlap is shown. The distribution of newly acquired ATRXΔRBR peaks to promoters and gene bodies is indicated in the bar graph. **c** Genome browser view of *Slc25a16* gene showing IgG (gray) and ATRX (purple) CUT&RUN tracks from WT ATRX and ATRXΔRBR cells. **d** Genome browser view of *Gins2* gene showing gain of ATRX signal (purple) in ATRXΔRBR. RNA Polymerase II (RNAPII, yellow) and H3K4me3 (green) ChIP-Seq tracks are shown. **e** Metaplot for RNAPII and H3K4me3 at TSS of ATRX peaks that are unique to WT MEFs (blue) and ATRX peaks that are unique to ATRXΔRBR (red). **f** Model ATRX binds RNAs that contain G-rich motifs and this prevents ATRX accumulation on chromatin (top). Loss of RNA interactions results in relocation of ATRXΔRBR to the G-rich DNA sequences on the gene body (bottom).

To identify regions of the genome that ATRX binds through RNA-dependent and independent mechanisms, we investigated the genome-wide localization patterns for WT ATRX and ATRXΔRBR using CUT&RUN^62,63^. Using this approach, we identified 9141 regions that showed ATRX enrichment in WT cells of which 54% were located within genes (14% TSS and 40% gene body) and 46% were intergenic (Fig. 4a). A similar analysis in ATRXΔRBR showed that the 6274 enriched regions were almost equally distributed between TSS (28%), gene body (35%), and intergenic regions (37%). We found that deletion of the RBR in ATRX resulted in the loss of ATRX from a number of regions (Figs. 4b, c). We conclude that at these sites ATRX depends on its RNA interactions for correct localization.

In our analysis of ATRX distribution in WT and ATRXΔRBR, we observed an increase in the proportion of TSS peaks in ATRXΔRBR (28%) compared with WT (14%). This increase in enrichment at TSS prompted us to examine the overlap between peaks in WT and ATRXΔRBR more closely. While there were a significant number of regions (5703) that lost ATRX upon RBR deletion (Fig. 4b), a number of regions retained ATRX (3438) suggesting that at these sites, ATRX may be tethered through RNA interactions mediated by the helicase domain or recruited through RNA-independent mechanisms. We also observed that a sizeable number of sites (2836) gained ATRX in ATRXΔRBR (Fig. 4b). Further analysis revealed that ATRXΔRBR protein was specifically redistributed to promoter regions that we defined as +/−2 kb surrounding the TSS (39%) and gene bodies (61%). There was no gain of ATRXΔRBR at intergenic sites. To our surprise, despite being compromised in RNA binding, ATRXΔRBR accumulates at promoters that contain RNA Polymerase II (Fig. 4d, e, and Supplementary Fig. 4a) and H3K4me3 (Fig. 4e), two indicators of active transcription. Analyses of Gro-seq data also show that genes that gain ATRXΔRBR have higher transcriptional output in WT (Supplementary Fig. 4b) as compared with genes that lose ATRX. These results suggest that while ATRXΔRBR becomes enriched at genes that are actively transcribed, its association to these sites is likely mediated by RNA independent interactions.

Since ATRX is known to bind tandem repeat DNA sequences, we tested whether the regions that gain ATRXΔRBR were enriched for specific DNA motifs. We compared regions that gain ATRX to those that lose it upon RBR deletion (Fig. 4b, gray and green areas) and found that ATRXΔRBR bound to DNA sequences that were GC-rich (Supplementary Fig. 4c), consistent with ATRX sequence preference. This preference for GC-rich sequences was also observed at promoter regions that gain ATRXΔRBR (Supplementary Fig. 4d). We propose that nascent RNAs prevents binding of WT ATRX at these “ectopic” sites that only appear when interactions with RNA are abolished by RBR deletion (Fig. 4f, top). When it is no longer able to bind RNA (ATRXΔRBR), ATRX instead binds the repetitive G-rich DNA at these sites (Fig. 4f, bottom). This is similar to a model proposed for PRC2 where its interaction with chromatin and RNAs is mutually antagonistic and functions to prevent PRC2 association and inappropriate silencing of specific genes^45,64^. Our findings indicate that RNAs function to target ATRX to certain genomic sites while also preventing its occupancy at regions with active transcription where ATRX function is not needed.

### ATRXΔRBR redistribution results in PRC2 complex recruitment

**Fig. 5:**
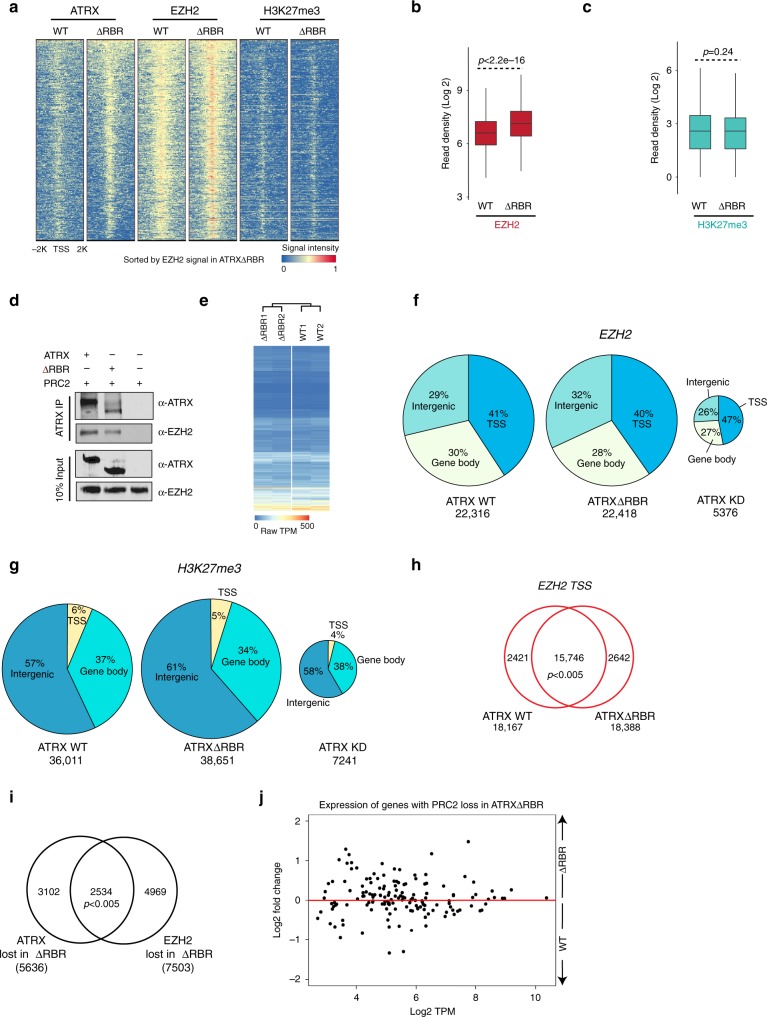
ATRXΔRBR accumulation at ectopic sites results in recruitment of PRC2 and results in derepression of a subset of PRC2 targets. **a** Heatmaps of ATRX, EZH2, and H3K27me3 signal across all TSS that acquire ATRX signal in ATRXΔRBR, sorted by ATRXΔRBR EZH2 signal. **b**, **c** Boxplots of read densities (Log2 scale) in WT ATRX and ATRXΔRBR for EZH2 (red) and H3K27me3 (teal) at ATRX peaks that are acquired at TSS in ATRXΔRBR. Each boxplot represents median (center line), interquartile range (box), and min-max range (whiskers). Read densities are computed over 2600 TSS that acquire ATRX in ATRXΔRBR. *P* values are calculated using two-sided Student’s *t* test. **d** Co-IP of ATRX, ATRXΔRBR, and PRC2 as indicated. Western blot detection using ATRX and EZH2 antibodies. Source data are provided as a Source Data file. Representative image from three independent experiments is shown. **e** Heatmap showing expression of genes that acquire ATRX peaks at TSS in ATRXΔRBR compared with their expression in WT ATRX cells. **f** EZH2 peak distribution in WT, ATRXΔRBR, and ATRX KD MEFs at TSS, gene body and intergenic sites. Total peak number is shown for each sample. **g** H3K27me3 peak distribution in WT, ATRXΔRBR and ATRX KD MEFs at TSS, gene body and intergenic sites. Total peak number is shown for each sample. **h** Venn diagram showing overlap between TSS that contain EZH2 in ATRX and ATRXΔRBR. Total number of peaks in each sample and their overlap is shown. *P* < 0.005, hypergeometric distribution. **i** Venn diagram showing overlap between genes that lose ATRX and EZH2 in ATRXΔRBR. Gene numbers in each sample and their overlap is shown. *P* < 0.005, hypergeometric distribution. **j** MA plot showing RNA-Seq TPM of 152 genes that lose EZH2 from TSS in ATRXΔRBR MEFs. A positive Log2 Fold Change indicates derepression in ATRXΔRBR.

Previous studies have shown that ATRX loss results in loss of PRC2 localization at polycomb target sites suggesting a dependence of PRC2 on the presence of ATRX at some sites^11^. We asked whether ATRXΔRBR binding to ectopic sites results in de novo PRC2 recruitment. We performed EZH2 and H3K27me3 CUT&RUN to evaluate their distribution in both WT and ATRXΔRBR. As a first step, we analyzed the genes that gain ATRX peaks in ATRXΔRBR. We found that in WT cells, these sites have very low levels of PRC2 occupancy. However, in ATRXΔRBR cells, there is an increased enrichment of EZH2 and H3K27me3 (Supplementary Fig. 5a, b). We observed this in two independent biological replicates (Supplementary Fig. 5a, b). Examining all TSS that gained ATRXΔRBR revealed that all these regions contained higher levels of ATRX and EZH2 as compared with the same sites in ATRX WT (Fig. 5a, b). Surprisingly, while H3K27me3 levels were modestly increased at some sites (Supplementary Fig. 5b), the overall levels of H3K27me3 did not show a significant increase at the new ATRX sites (Fig. 5a, c). Therefore, ectopic recruitment of ATRXΔRBR resulted in increased PRC2 occupancy.

ATRX and PRC2 are known to interact through a region within ATRX that overlaps the RBR^65^. To determine whether PRC2 recruitment to ectopic sites could be through its interactions with ATRX, we tested if PRC2 interacts with ATRXΔRBR in vitro. Immunoprecipitation experiments showed that deletion of the RBR did not alter the ability of ATRX to bind PRC2 (Fig. 5d), suggesting that ATRXΔRBR localization to ectopic sites can directly recruit PRC2. Next, we analyzed the effect of ATRX redistribution and consequent PRC2 recruitment on gene expression levels. Comparison of gene expression in WT and ATRXΔRBR showed that out of 15,614 genes, 973 were differentially expressed in ATRXΔRBR. Surprisingly, we found that most of the 1847 genes that acquired ATRXΔRBR and PRC2 did not show an obvious change in expression (Fig. 5e), suggesting that this newly recruited PRC2 was not able to deposit H3K27me3. We compared the degree of PRC2 accumulation at these new sites to PRC2 levels at bona fide polycomb targets, defined as those containing peaks of EZH2 enrichment. While EZH2 accumulation at the new sites almost reached levels seen at polycomb targets (Supplementary Fig. 5d), we found that H3K27me3 accumulation did not (Supplementary Fig. 5e). These findings are consistent with an inhibitory effect of nascent RNAs from active transcription sites on the catalytic activity of PRC2^20,22,66–68^.

### RBR deletion results in PRC2 loss at some polycomb targets

To gain a genome-wide view on how deletion of ATRX–RNA binding alters PRC2 localization, we analyzed EZH2 and H3K27me3 distribution in WT and ATRXΔRBR MEFs. As a control, we used ATRX knockdown (ATRX KD) cells that have previously been shown to lose EZH2 enrichment at PRC2 target genes. Analysis of peak distribution in WT MEFs showed that EZH2 peaks (22,316) were almost equally distributed between transcription start sites (TSS, 41%), gene bodies (30%) and intergenic sites (29%) (Fig. 5f). In ATRXΔRBR, the number of enriched regions (22,418) and their distribution was very similar to WT cells with 40% at TSS, 28% at gene bodies and 32% intergenic sites (Fig. 5f). Consistent with previous studies, ATRX KD resulted in a significant reduction in EZH2 enriched regions (5376). H3K27me3 also showed similar trends with the number of enriched sites not showing large differences when we compared ATRX WT to ATRXΔRBR (36,011 in ATRX WT and 38,651 in ATRXΔRBR). The distribution of H3K27me3 peaks in TSS, gene body and intergenic regions was also not different between ATRX WT and ATRXΔRBR (Fig. 5g). Similar to EZH2, ATRX KD cells showed a significant loss of H3K27me3 enrichment at all sites (Fig. 5g). Further analysis of global changes in gene expression between ATRX WT, ATRXΔRBR, and ATRX KD showed that at both non-polycomb target genes and polycomb target genes, ATRXΔRBR did not show obvious derepression. In comparison, in ATRX KD, both these groups of genes were upregulated to a greater extent (Supplementary Fig. 6a, b).

Next, we examined how loss of ATRX–RNA binding impacted PRC2 recruitment. First, we determined the overlap between ATRX and PRC2 peaks in WT. We found that ATRX and PRC2 shared occupancy at a large number of peaks (5969) (Supplementary Fig. 6c) and there was positive correlation between the datasets (Supplementary Fig. 6d). We examined PRC2 distribution at TSS in WT and ATRXΔRBR and found that the majority of EZH2 sites identified in WT cells retained EZH2 in ATRXΔRBR (Fig. 5h). Only a small fraction of TSS sites (2421) lost EZH2 in ATRXΔRBR (Fig. 5h). A gene level analysis showed a significant overlap between genes that lose ATRX and EZH2 in ATRXΔRBR (Fig. 5i). We examined the expression of polycomb genes that lose or gain EZH2 in ATRXΔRBR, and found that genes that lose EZH2 are more likely to be derepressed by twofold or more as compared with genes that gain EZH2 (Fisher’s exact test, *P* = 0.029) (Fig. 5j). We tested if the extent of derepression observed at some polycomb target genes upon loss of ATRX–RNA interactions is comparable to loss of PRC2. We examined expression of Parp12 and Rnf40, two polycomb target genes in WT MEFs, ATRXΔRBR, and MEFs treated with an EZH2 histone lysine methyltransferase inhibitor, Tazemetostat^69^. We found that both Parp12 and Rnf40 are upregulated to the same extent in ATRXΔRBR and in WT MEFs after treatment with EZH2 inhibitor (Supplementary Fig. 6e). As expected, expression levels of Actin, a non-polycomb target is unchanged. Thus ATRX–RNA interactions regulate repression of a subset of polycomb target genes.

## Discussion

ATRX is an RNA-binding protein that functions in many biological processes including X chromosome inactivation, polycomb repression, heterochromatin maintenance and telomere protection. While ATRX in complex with the DAXX histone chaperone and their function in histone H3.3 deposition has been studied to an appreciable extent, the role of its RNA interactions remains less clear. ATRX interacts with Xist lncRNA on the Xi and this interaction plays a role in regulating PRC2 enrichment on the Xi^11^. ATRX also binds the muscle specific ChRO1 lncRNA and this interaction is important for the deposition of the histone variant H3.3 at satellite repeats and organization of constitutive heterochromatin^41^. On the contrary, previous studies have shown that ATRX interactions with the TERRA non-coding RNA antagonize ATRX localization to telomeres^5^. In these studies, knockdown of TERRA result in increased presence of ATRX at telomere. These examples highlight the divergent roles of ATRX–RNA interactions in specific contexts. While these individual examples provide insight into molecular mechanisms of ATRX in specific processes, they do not query general roles of RNA interactions in the genome-wide targeting and function of ATRX.

Here, we identify and disrupt the ATRX–RBR that allowed us to directly interrogate the functional consequence of loss of ATRX–RNA interactions. Using a cross-linking and mass spectrometry approach we identify RBRs across ATRX. We found a major RBR that is harbored in the N terminal of the protein and is distinct from its histone binding and chromatin remodeler domains (Fig. 2). Deletion of the RBR significantly destabilizes ATRX–RNA interactions in vitro and in vivo without affecting protein stability (Figs. 2 and 3). ATRX that is defective in RNA binding (ATRXΔRBR) showed diminished localization to chromatin (Fig. 3). ATRX localization to chromatin is not entirely dependent on its interactions with RNA. Other targeting mechanisms may include interactions with other proteins, DNA, and modified histones (H3K9me3) that are recognized by the PHD fingers at the N terminus of the protein. Our studies show that loss of RNA interactions results in loss of ATRXΔRBR from some ATRX target genes and gain at other sites that correlate with genes that are actively transcribed (Fig. 4). In agreement with our finding, a recent study showed that in cancer cell lines that harbor ATRX deletions that result in loss of the RNA binding region, the mutant ATRX relocates to active gene promoters^70^. In our study, the gain of ATRX at these sites is correlated with increased PRC2 complex occupancy (Fig. 5). These newly established ATRX/PRC2 sites do not accumulate the same extent of PRC2 or H3K27me3 as bona fide PRC2 target sites, even though the interaction between PRC2 and ATRXΔRBR is not compromised. This suggests that PRC2 activity and stable association to specific sites could be regulated by additional factors. Our results point to a model where at some sites ATRX–RNA-binding functions to recruit ATRX, and at other regions functions antagonistically to prevent ectopic ATRX localization. Several studies have shown that the localization of the PRC2 complex is similarly altered by its ability to interact with distinct RNAs^2^. Our findings also suggest that the localization of the PRC2 complex to many regions is not dependent on the interaction between ATRX and RNA, but rather the presence or absence of ATRX at these sites. Consistently, regions of the genome that contain ATRX overlap significantly with those that contain EZH2. We find that ATRX–RNA interactions regulate the recruitment of PRC2 to only a subset of polycomb target sites that become derepressed in response to PRC2 loss (Fig. 5).

ATRX is a highly mutated protein in many cancers^6,71–74^. Numerous missense mutations in ATRX have been discovered by whole-exome sequencing of cancers and these reside in the region of ATRX that we have found is able to regulate RNA interactions. Indeed, small deletions and point mutations within this region significantly reduce ATRX localization to heterochromatin^75,76^ and it is therefore conceivable that the ATRX localization defects observed in these mutants may be due to deficiencies in RNA binding.

ATRX localizes to repetitive areas of the genome such as centromeric and pericentromeric heterochromatin, the Xist gene and other variable number tandem repeats. A common theme in all these regions is their ability to produce repetitive RNAs. Dysregulation of repetitive elements is common in many cancers. Recent studies have shown that some fusion oncoproteins can cause transcription of specific microsatellite repeats that can adopt capabilities of active enhancers^77^. Further studies directed toward identifying the RNA interactome of ATRX and uncovering the structural and molecular mechanisms utilized for specific transcript recognition will provide clues to understanding how ATRX is recruited. This can reveal pathways that are deregulated in cancers with compromised ATRX function that can then be exploited in the development of novel therapies.

## Methods

### Cell lines and cell culture

Female mouse embryonic fibroblast cells (MEFs) (ATRX WT, KO, ΔRBR, and KD) were cultured in DMEM supplemented with 10% FCS (GemCell U.S. Origin SuperCalf Serum #100-510). Parental MEFs were obtained from the laboratory of Dr. Jeannie T. Lee. ATRX KD MEFs were generated using shRNA^11^, selected with puromycin. *Spodoptera frugiperda* (Sf9) insect cells were obtained from Expression Systems and grown in Expression Systems ESF 921 media (Fisher #NC9541308) or HyClone CCM3 media (GELifeSciences #SH30065.02) at 27 °C.

To generate ATRXΔRBR MEF lines with CRISPR/cas9, two ATRX guide RNAs flanking the RBR were cloned into pX459 (pSpCas9(BB)-2A-Puro) and transfected into MEFs. Genomic DNA from potential ATRXΔRBR clones was isolated using QuickExtract (Epicentre # QE09050). Briefly, once cells were confluent, cell culture media was removed, 25 µl of QuickExtract (ice cold) was added and incubated on ice for 5 min. After incubation, the cells were resuspended in the QuickExtract solution and transferred to a PCR tube. The samples were heated at 65 °C for 6 min followed by 98 °C for 2 min. One to two microliters of genomic DNA were used to amplify the region of interest by PCR using Taq polymerase (Bioline # BIO-25042). Fragments with a potential deletion were sequenced.

MEF cells were treated with 3 μM EZH2 inhibitor (Tazemetostat EPZ-6438) for 5 days. RNA was extracted with Trizol (Thermo Fisher #15596018) and processed for reverse transcription reaction as per the manufacturer’s instructions at 37 °C for 1 h, and then 1 µg RNA was used for reverse transcript with random primer. Primers used are listed in Supplementary Table 1.

### Nuclear fractionation

Forty million cells were used for each fractionation. Cells were washed with 2 ml Buffer A (10 mM HEPES pH 7.9, 5 mM MgCl_2_, and 0.25 M sucrose) per 10 million cells. Fresh 0.5 mM DTT, 0.2 mM PMSF, and 50× protease inhibitors were added and the cells were centrifuged at 2500 × *g* for 5 min at 4 °C. The supernatant was discarded, and the pellet resuspended in 500 µl Buffer A containing 0.2% NP-40 per 10 million cells and lysed on ice for 5 min. The cells were spun down at 2500 × *g* for 5 min at 4 °C and the supernatant was collected (cytosolic extract). The pellet was resuspended in 1 ml of Buffer A without detergent. Two hundred microliters (20%) was collected, spun down and resuspended in BC500 buffer (25 mM Tris pH 7.6, 0.2 mM EDTA, 500 mM KCl, 10% glycerol) and treated as the nuclear extract plus nuclear pellet fraction. The remaining 800 µl were split into two tubes with 400 µl each, spun down at 2500 × *g* for 5 min at 4 °C and pellets resuspended in 160 µl of Buffer B containing 300 mM NaCl (10 mM HEPES pH 7.9, 0.1 mM EDTA, 1.5 mM MgCl_2_, and 25% glycerol). Ten micrograms of RNase A (Roche Boehringer Mannheim cat#10109169001) was added to one of the tubes and both were incubated on ice for 60 min. The nuclei were spun down at 18,000 × *g* for 15 min and the supernatant was collected and labeled nuclear extract. The nuclear pellet was resuspended in 100 µl of BC1000 (25 mM Tris pH 7.6, 0.2 mM EDTA, 1000 mM KCl, 10% glycerol), incubated on ice for 10 min, and sonicated. Four hundred microliters of BC0 (25 mM Tris pH 7.6, 0.2 mM EDTA, 10% glycerol) was added to reduce the salt concentration. The combined nuclear extract and pellet fraction were sonicated and centrifuged at 10,000 × *g* for 15 min to remove cell debris. A 6% SDS PAGE gel was used to run 5% of each fraction for western blot.

### Immunofluorescence

Immunofluorescence was performed according to Zhang et al.^78^ with minor modifications as described below. For RNase A treatment, after CSKT treatment the cells were treated with 1 mg/ml or 100 μg/ml RNase A diluted in PBS at room temperature for 10 min prior to fixation with 4% paraformaldehyde. Antibodies used are listed in Supplementary Table 2.

### ATRX antibody generation and purification

A fragment of human ATRX protein corresponding to nucleotides 7072–7671 was cloned into pet101 (Invitrogen Cat#K10101) following manufacturer’s instructions. ATRX protein was expressed in BL21 star (Thermo Fisher cat#C601003) using standard protein expression procedures and purified using Ni-NTA agarose (Qiagen cat#30210) under native conditions. Purified protein was dialyzed into 1× PBS and used for antibody generation (Cocalico Biologicals, Inc).

ATRX antibodies were affinity purified from serum. Briefly, purified ATRX-His antigen was coupled to NHS-activated agarose (Pierce cat#26200) according to manufacturer’s instructions. Antigen coupled beads and 1 ml of serum were incubated overnight at 4 °C on a rotating wheel. Beads were washed 2× with 4 column volumes (CV) PBS, 2× with 4CV PBS containing 0.5 M NaCl, and 1× with 4CV PBS. The antibody was eluted with 5CV (1CV at a time) of 0.1 M glycine pH 2.5 directly into tubes containing 10% volume of 1.5 M Tris pH 8.8. Glycerol was added to purified antibody and aliquots were stored at −20 °C.

### Protein purification

Full-length FLAG-ATRX-HA, FLAG-ATRXΔRBR-HA, and FLAG-ATRX helicase domain were generated using NEBuilder (NEB #E2621S). Proteins were purified from Sf9 cells^11^ using M2 agarose beads (Sigma #M8823-1ML). GST-RBR and GST proteins were purified from *E. coli* using Glutathione Agarose 4B beads (Affymetrix cat#78820) according to manufacturer’s instructions.

### RNA EMSA

EMSAs were performed according to Cifuentes-Rojas et al.^20^. Briefly, in vitro transcribed RNAs were end-labeled with T4 PNK. Reactions were assembled with purified protein and labeled RNAs in binding buffer containing 50 mM Tris pH 8.0, 100 mM NaCl, 5 mM MgCl_2_, 10 μg/ml BSA, 0.05% NP-40, 1 mM DTT, 20 U RNasin (Promega), and 5% glycerol in a final volume of 20 μl. A total of 50 ng/μl yeast tRNA (Ambion cat# AM7119) was used as a nonspecific competitor. Reactions were incubated at 30 °C for 30 min and loaded onto a 0.4% high-strength agarose (Sigma) gel in THEM buffer (66 mM HEPES, 34 mM Tris, 0.1 mM disodium EDTA, and 10 mM MgCl_2_) and run for 90 min at 90 V at 4 °C. Gels were dried and exposed to phosphorimager screens.

### CLIP

CLIP was performed according to Jeon and Lee^59^. Briefly, MEFs UV cross-linked at 254 nm (2000 J/m^2^) in ice-cold PBS. Cross-linked cells were lysed in PBS containing 0.5% NP-40, 0.5% sodium deoxycholate, 400 U/ml Rnasin, and protease inhibitor cocktail (Sigma) at 4 °C for 25 min, followed by Turbo DNase treatment. Supernatant was incubated with 5 μg of IgG or ATRX antibodies immobilized on Dynabeads Protein G, overnight. Beads were washed three times with PBS containing 1% NP-40, 0.5% sodium deoxycholate, and additional 150 mM NaCl (total 300 mM NaCl), and treated with Turbo DNase (10 U) for 30 min. Beads were washed three more times with wash buffer supplemented with 10 mM EDTA, and incubated in 100 mM Tris-HCl (pH 7.5), 50 mM NaCl, 10 mM EDTA, 100 μg of Proteinase K (Roche), and 0.5% SDS for 30 min at 55 °C. RNA was recovered by Trizol extraction, processed for reverse transcriptase reactions and qPCR analysis.

### In vitro RNA/DNA immunoprecipitation

MBP DNA and Xist Repeat A DNA were amplified by PCR. MBP RNA and Xist RNA were transcribed in vitro (MEGAscript T7 #AM1334), treated with Turbo DNase (10 U) for 5 min and purified using Trizol. Five micrograms of MBP or Xist Repeat A RNA (or DNA in the case of DNA-IP) and 2 µg of protein were incubated in binding buffer (2 mM MgCl_2_, 100 U/ml Rnase Inhibitor, 0.1 mg/ml yeast tRNA, 0.05% BSA, 0.2% NP-40) at 4 °C overnight. GST-RBR and GST proteins were incubated with Glutathione Agarose 4B beads (Affymetrix #78820). FLAG-ATRX-HA, FLAG-ATRXΔRBR-HA were incubated with FLAG agarose beads (Sigma #A2220). Beads were washed sequentially with binding buffers containing 150 mM NaCl and 15 mM NaCl. Bound RNAs were extracted with Trizol and reverse transcribed. For preferential binding experiments, 5 μg MBP RNA and 5 μg Xist Repeat RNA were mixed and incubated with 2 μg GST-RBR or GST proteins. For comparison of interactions between the helicase domain or RBR with Repeat A RNA interactions, 25 pmol of protein was incubated with 5 μg RNA for binding reactions.

Total RNA was extracted from MEF cells with Trizol. Five micrograms of total RNA and 2 µg of FLAG-ATRX-HA, FLAG-ATRXΔRBR-HA were incubated with FLAG agarose beads (Sigma #A2220) in binding buffer (2 mM MgCl_2_, 100 U/ml RNase Inhibitor, 0.05% BSA, 0.2% NP-40) at 4 °C overnight. Beads were washed sequentially with binding buffers containing 150 mM NaCl and 15 mM NaCl. Bound RNAs were extracted with Trizol and visualized by SYBR Gold staining.

### Co-immunoprecipitation

For endogenous IP, 1 mg nuclear extract was incubated with ATRX antibody coupled to protein A agarose beads. Bound proteins were washed with BC300 buffer and eluted with 0.1 M Glycine (pH 2.5). For immunoprecipitation using purified proteins, 0.5 μg PRC2 and 2 μg FLAG-ATRX-HA, FLAG-ATRXΔRBR-HA were incubated with ATRX antibody and 20 μl Dynabeads protein G beads (Invitrogen #10004D) in BC-100 at 4 °C overnight. Beads were washed with BC300 and bound proteins were eluted with 0.1 M Glycine (pH 2.5). For IP with HA-tagged ATRX, WT ATRX-HA or ATRXΔRBR-HA were expressed in HEK293 cells. 0.5 mg nuclear extract from each was incubated with 20 μl HA beads (Sigma #A2095) at 4 °C overnight. Beads were washed with BC300 and bound proteins were eluted by heating at 95 °C for 10 min in 1×SDS loading buffer.

### In vivo RBR-ID

In vivo RBR-ID data for ATRX was taken from He et al.^39^. Peptides corresponding to ATRX were manually aligned against canonical ATRX isoform to account for multiple isoforms reported in the original dataset by the automatic isoform matching process performed by the mass spectrometry analysis software, then normalized and quantified^39^.

### In vitro RBR-ID

Four hundred nanograms of a 192-nucleotide fragment of Repeat A RNA was refolded for 20 min at 25 °C in 1× refolding buffer [10 mM bis-tris (pH 6.7), 10 mM KCl, 3 mM MgCl_2_]. RNA was incubated with 500 ng purified ATRX protein on ice in 1× binding buffer [10 mM HEPES (pH 7.2), 100 mM KCl, 3 mM MgCl_2_, 5% glycerol, 1 mM DTT]. RNA-protein mixtures were cross-linked with 1 J/cm^2^ UVB radiation (312 nm) using Spectrolinker XL-1500 (Spectroline). Cross-linked complexes were diluted in trypsin digestion buffer (final: 50 mM NH_4_HCO_3_ [pH 8], 1 mM MgCl_2_, 1 mM CaCl_2_), and treated with 12.5 μg/mL Rnase A for 30 min at 37 °C. To reduce proteins, samples were treated with 5 mM dithiothreitol for 60 min at 25 °C, then cysteines were alkylated with 14 mM iodoacetamide for 30 min in the dark, before additional reduction with 5 mM dithiothreitol for additional 15 min in the dark. Proteins were digested with trypsin at a protease:sample ratio of 1:20 and incubated overnight at 37 °C. Peptides were quenched with formic acid and desalted on C18 stage tips, then dried and resuspended in 0.1% trifluoroacetic acid followed by mass spectrometry analysis^39^.

### Mass spectrometry

Samples were analyzed by using a nanoLC-MS/MS setup. nanoLC was configured with a 75 µm ID × 20 cm Reprosil-Pur C18-AQ (3 µm; Dr. Maisch GmbH, Germany) nano-column using an EASY-nLC nanoHPLC (Thermo Scientific, San Jose, CA, USA). The HPLC gradient was 0–30% solvent B (A = 0.1% formic acid; B = 95% acetonitrile, 0.1% formic acid) over 30 min followed by 5 min from 30 to 85% B and 10 min at isocratic 85% B. The flowrate was set to 300 nL/min. nLC was coupled with either an Orbitrap Fusion Tribrid mass spectrometer (four replicates) or an Orbitrap Elite mass spectrometer (four replicates) (Thermo Fisher Scientific, San Jose, CA, USA). Spray voltage was set at 2.5 kV and capillary temperature was set at 275 °C. For samples run on the Orbitrap Fusion instrument, samples were acquired using a data-dependent acquisition (DDA) method or a data-independent acquisition (DIA) method. All scans were performed in the orbitrap mass analyzer. The full MS scan was acquired at 120,000 (DDA) or 60,000 (DIA) resolution using the *m/z* range 300–1100, while the MS/MS scans were performed at a resolution of 15,000 using a higher-energy collisional dissociation (HCD) set to 27. For DDA, the cycle time was set to 2 s. For DIA, we used isolation windows of 20 *m*/*z* spanning between the *m/z* range 300–1100 and multiplexed in groups of 4, for a total of 10 MS/MS scans per cycle (40 windows multiplexed in groups of 4). Samples run on the Orbitrap Elite instrument were processed as above but instead with a 45-min HPLC gradient, then acquired using a canonical DDA method. Full MS scan was performed in the orbitrap, while the MS/MS scans were performed for the top 10 most intense signals in the ion trap using a collisional-induced dissociation (CID) set to 35.

DDA raw files were processed through the MaxQuant program^79^. All parameters for database searching were kept as default. We used a custom database containing common contaminants, human ATRX (UniProt accession P46100) and bovine Rnase1 (P61823).

The “Intensity” value of the peptides from MaxQuant were used for quantification. For DIA runs, we extracted the chromatograms based on the spectral library generated by the MaxQuant search of DDA runs. The extracted ion chromatogram was performed by using Skyline^80^. For quantification, we used the area under the curve of the precursor signal.

### RBR-ID analysis

After removal of suspected contaminants, Maxquant peptide abundances were normalized by the sum of all peptide intensities in each MS run. For each peptide a log2-converted ratio was calculated between samples treated with or without 4SU to assess depletion mediated by RNA cross-linking. For generation of these ratios, the mean intensity of peptides with only zero values was assigned as 0.5*(minimum observed mean peptide intensity). *P* values for peptides were calculated using an unpaired, two-sided Student’s *t* test. RBR-ID scores, which are an estimate of RNA cross-linking likelihood^39^, were calculated for each peptide as follows:

$${\mathrm{score}} = - {\mathrm{log}}_{\mathrm{2}}\left( { + {\mathrm{4SU}}/\!\! -\! {\mathrm{4SU}}\,{\mathrm{normalized}}\,{\mathrm{peptide}}\,{\mathrm{ratio}}} \right)\ast \left( {{\mathrm{log}}_{{\mathrm{1}}0}\left( {P{\hbox{-}}{\mathrm{value}}} \right)} \right)^{\mathrm{2}}$$

For residue level RBR-ID scores, we summed the RBR-ID score of each peptide overlapping any given amino acid and smoothed the resulting curve using Friedman’s ‘super smoother’, a variable span smoother (https://stat.ethz.ch/R-manual/R-devel/library/stats/html/supsmu.html) (DTIC document).

### CUT&RUN and RNA-sequencing

CUT&RUN was performed exactly as described in^62,63^ using EZH2(Cell signaling #5246S), H3K27me3 (Active motif #61017) and ATRX antibodies. DNA was end-repaired using End-It Repair Kit, tailed with an A using Klenow exo minus, and ligated to adapters (NEB #E7600S) with T4 DNA ligase. Fragments >150 bp were size-selected with AMpure beads (Beckman Coulter) and subjected to ligation-mediated PCR amplification with barcoded adapters (NEB #E7600S) for Illumina sequencing using Q5 DNA polymerase. All enzymes except Q5 (NEB) were from Enzymatics (a Qiagen company). PolyA RNA (NEB # E7490S) was isolated from two biological replicates and libraries prepared using the Ultra II directional RNA library prep kit for illumina (NEB #E7760S). Sequencing was performed on a NextSeq 500 (Illumina).

### Sequencing analysis

Raw reads were mapped to the mouse genome (mm10) with Bowtie2 version 2.2.9^81^ using default parameters. Peaks were called for each sample using MACS2^82^ using the parameters: --broad --broad-cutoff 0.1. GRO-seq raw data were downloaded from GSM665997^83^. H3K4me3 ChIP-Seq raw data were downloaded from GSM723006^84^. RNA Polymerase II ChIP-Seq raw data were downloaded from GSM1887380^85^. RNA-Seq data were aligned using STAR version 2.6.1^86^. RSEM^87^ was used to obtain estimated counts and TPM (transcripts per kilobase per million). CUT&RUN heatmaps were generated using ChAsE^88^. RNA-Seq TPM heatmaps were generated using pheatmap version 1.0.12. Metaplots were generated using Deeptools. For TPM heatmaps and scatterplots, only genes with at least 5 TPM per sample were considered. Differential analysis of RNA-Seq data were performed using DESeq2 version 1.24.0^89^. For RNA-Seq differential analysis, only genes with more than 10 total estimated counts across all samples were considered.

### Quantification and statistical analysis

All experiments were performed at least three times. *P* values were calculated using student’s *t* test. Statistical analyses were performed using RStudio. Plots were generated using ggplot2. Bedtools version 2.26.0 was used for genomic analyses^90^.

### Reporting summary

Further information on research design is available in the Nature Research Reporting Summary linked to this article.

## Supplementary information

Supplementary Information

Reporting Summary

## Data Availability

Sequencing data generated for this study have been deposited in the NCBI GEO as GSE130452. The mass spectrometry proteomics data have been deposited to the ProteomeXchange Consortium via the PRIDE [1] partner repository with the dataset identifier PXD017806 and 10.6019/PXD017806. The source data underlying Figs. 1b–h, 2b–h, 3a–i, 5d, and Supplementary Figs. 1a–c, 2d, 3c, and 6e are provided as a Source Data file. All data is available from the corresponding author upon reasonable request.

## Source Data

Source Data
