# Supplementary material for: Disruption of ATRX-RNA interactions uncovers roles in ATRX localization and PRC2 function
Source: Nat Commun. 2020 May 6;11:2219. doi: 10.1038/s41467-020-15902-9 (PMC7203109; doi:10.1038/s41467-020-15902-9)
Supplement: Supplementary file 1 — Supplementary Information [file 41467_2020_15902_MOESM1_ESM.pdf]

SUPPLEMENTARY INFORMATION

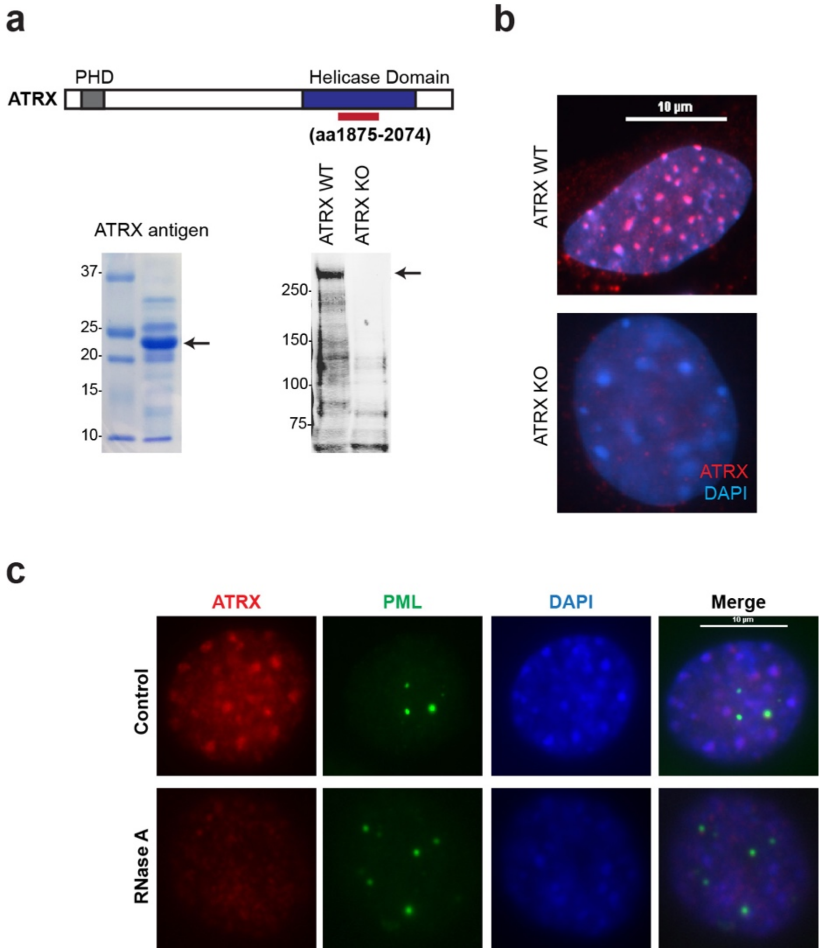

Supplementary Figure 1. Characterization of ATRX antibodies.

a) Top- schematic for location of ATRX amino acid residues used for production of an ATRX polyclonal antibody, highlighting PHD fingers (grey) and helicase domain (dark blue). Bottom left- Coomassie stain of ATRX antigen (indicated with arrow). Bottom right- western blot for ATRX using affinity purified antibody in MEF nuclear extract as indicated on top. Expected location of full length ATRX is indicated with an arrow. Source data are provided as a Source Data file. Representative image from 6 independent experiments is shown.

b) Immunostaining of WT and ATRX KO MEFs with affinity purified ATRX antibodies (red). Source data are provided as a Source Data file. Representative image from 2 independent experiments is shown. Scale bar = 10μm

c) Immunostaining of control (top) and RNase A treated (bottom) MEFs with ATRX (red) and PML protein (green) antibodies. Nucleus is stained with DAPI. Source data are provided as a

Source Data file. Representative image from 2 independent experiments is shown. Scale bar = 10μm

a

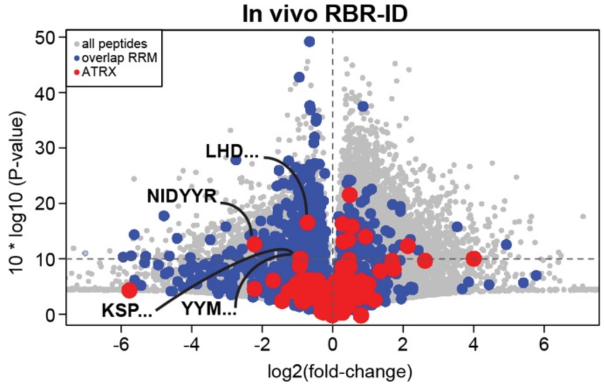

sequence	start	end	logFold.312	pval.312
LHDFLAHSSEESEETCSSPR	18	37	-0.7040669	0.02125196
YYMSDDISRDSDGMDEQCR	202	220	-0.8883163	0.09567925
KSPGSGCILAHCMGLGK	1569	1585	-0.9153704	0.09401193
NIDYYR	2063	2068	-2.2033943	0.05227072

b

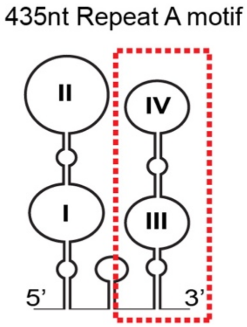

c

In vitro RBR-ID

sequence	start	end	log2fold	p.value
EHKVIDAK	431	438	-2.101503	0.018
EHKVIDAKFETK	431	442	-4.539131	0.038
DISKSEAKLSR	456	466	-3.407408	0.092
QKLSVPVR	689	696	-1.479375	0.004
QSETVDQNSDSDEMLAILK	721	739	-1.325696	0.074
KLKEIFNK	1699	1706	-3.596318	0.010

d

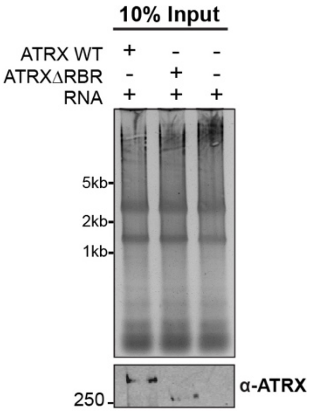

Supplementary Figure 2. Identification of ATRX RNA binding region.

a) Top- Volcano plot showing log fold changes in peptide intensities on the x axis and p values on the y axis for ± 4SU. All peptides are shown in grey, peptides overlapping annotated RRM domains are in blue and ATRX peptides are in red. Bottom- ATRX RNA binding peptides identified in in vivo RBR-ID experiments. P values are calculated using two-sided Student's t test.

b) Schematic of one possible structure of the 435nt fragment of Xist Repeat A RNA[1].

c) Sequences of peptides that are most significantly depleted in all biological and technical replicates from in vitro RBR-ID experiments. P values are calculated using two-sided Student's t test.

d) 10% input of RNA (top) and ATRX, ATRXΔRBR proteins (bottom) used for total RNA IP. Source data are provided as a Source Data file. Representative image from 3 independent experiments is shown.

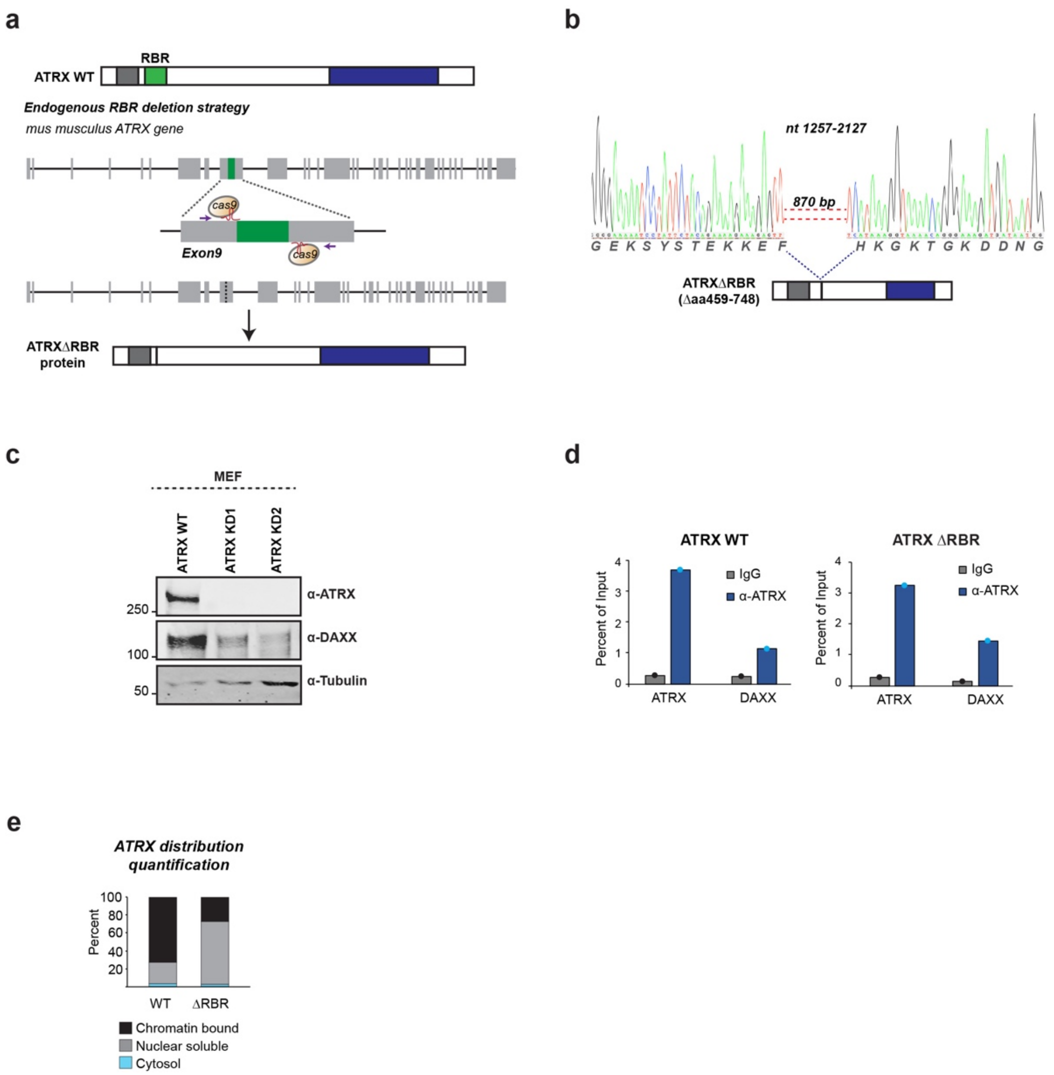

Supplementary Figure 3. Generation of ATRXΔRBR by CRISPR.

a) Schematic of ATRX protein. RBR (green), PHD fingers (grey), and helicase domain (dark blue). CRISPR Cas9 strategy for RBR deletion at the endogenous mouse *ATRX* gene. Location of gRNAs (red) and screening primers (purple) flanking the RBR within exon 9 are shown.

b) Protein sequence from ATRXΔRBR. Exact nucleotides (corresponding to mATRX cDNA) and amino acids deleted in the ATRXΔRBR clone are shown.

c) Western blot for ATRX, DAXX, and Tubulin in WT and ATRX KD MEF nuclear extract**.** Source data are provided as a Source Data file. Representative image from 4 independent experiments is shown.

d) Quantification of endogenous ATRX and DAXX immunoprecipitation from WT and ATRXΔRBR MEFs using anti ATRX antibodies. This graph represents quantification of the western blot signal from Fig. 3B.

e) Quantification of ATRX and ATRXΔRBR distribution in cytosol, nuclear soluble and chromatin bound fractions. This graph represents quantification of the western blot signal from Fig. 3H.

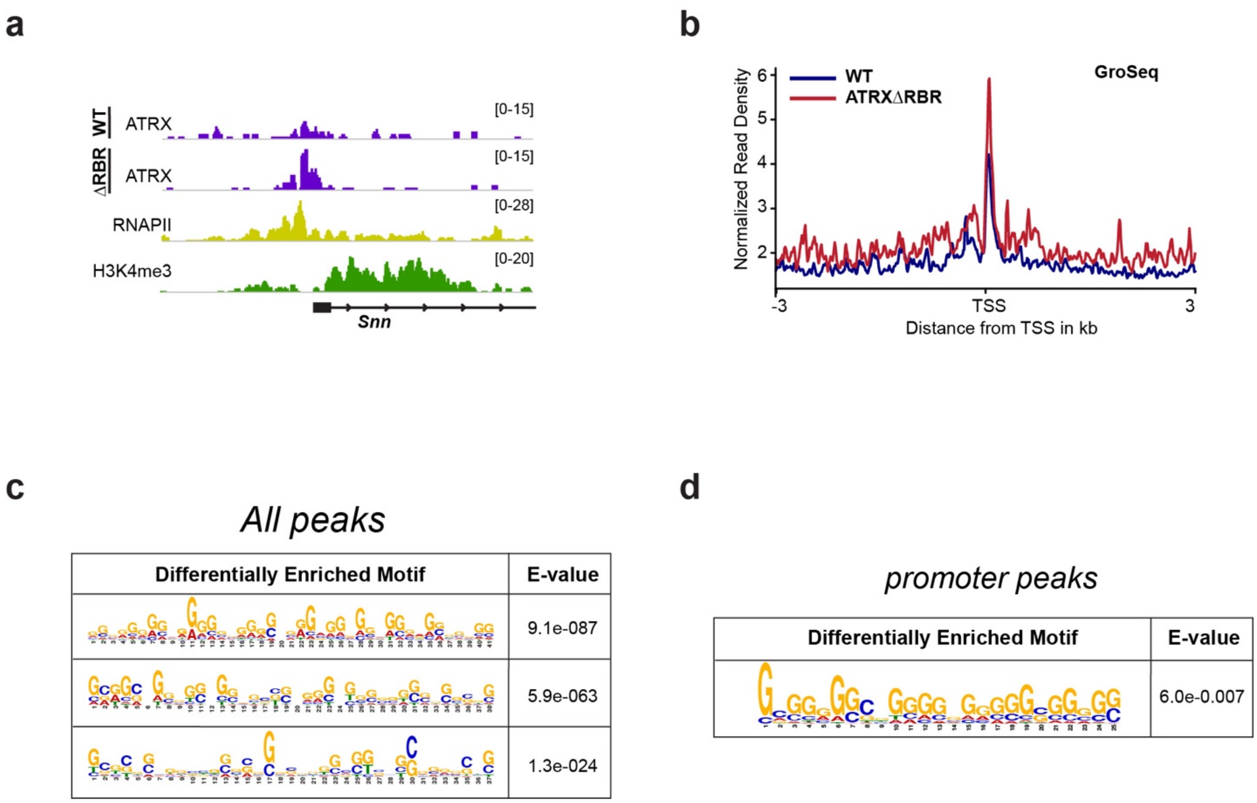

Supplementary Figure 4. ATRXΔRBR accumulates at G rich regions within actively transcribed genes.

a) Genome browser view of *Snn* gene showing gain of ATRX signal (purple) in ATRXΔRBR. RNA Polymerase II (RNAPII, yellow) and H3K4me3 (green) ChIP-Seq tracks are shown.

b) Metaplot for Gro-Seq at TSS of ATRX peaks that are unique to WT MEFs (blue) and ATRX peaks that are unique to ATRXΔRBR (red).

c) Significantly enriched motifs found at all ATRX peaks that are unique to ATRXΔRBR compared to all ATRX peaks that are unique to WT MEFs.

d) Significantly enriched motifs found at ATRX promoter peaks (+/- 2kb from TSS) that are unique to ATRXΔRBR compared to ATRX promoter peaks that are unique to WT MEFs.

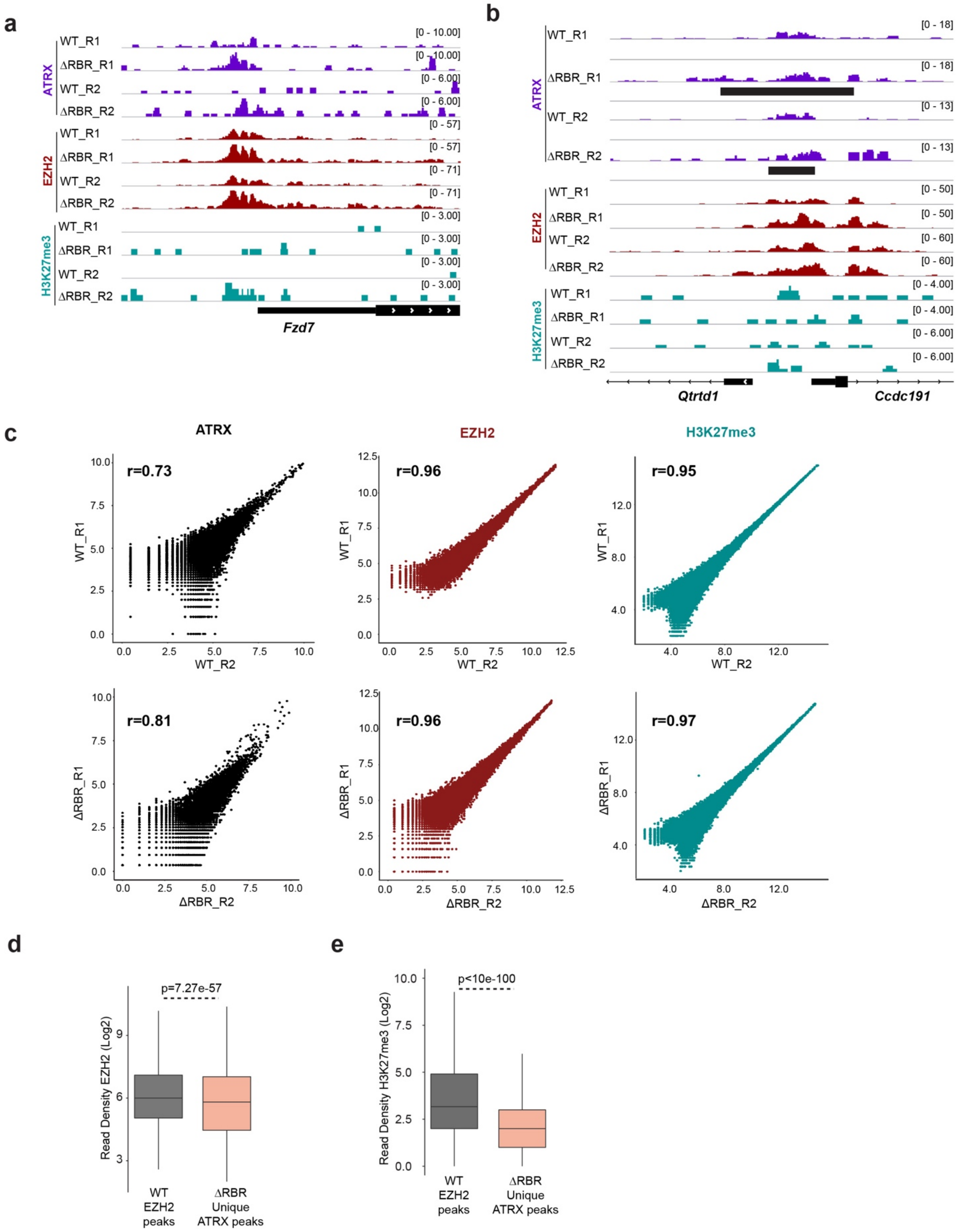
a
ATRX
WT_R1
ΔRBR_R1
WT_R2
ΔRBR_R2
[0 - 10.00]
[0 - 10.00]
[0 - 6.00]
[0 - 6.00]
EZH2
WT_R1
ΔRBR_R1
WT_R2
ΔRBR_R2
[0 - 57]
[0 - 57]
[0 - 71]
[0 - 71]
H3K27me3
WT_R1
ΔRBR_R1
WT_R2
ΔRBR_R2
[0 - 3.00]
[0 - 3.00]
[0 - 3.00]
[0 - 3.00]
Fzd7
b
ATRX
WT_R1
ΔRBR_R1
WT_R2
ΔRBR_R2
[0 - 18]
[0 - 18]
[0 - 13]
[0 - 13]
EZH2
WT_R1
ΔRBR_R1
WT_R2
ΔRBR_R2
[0 - 50]
[0 - 50]
[0 - 60]
[0 - 60]
H3K27me3
WT_R1
ΔRBR_R1
WT_R2
ΔRBR_R2
[0 - 4.00]
[0 - 4.00]
[0 - 6.00]
[0 - 6.00]
Qtrtd1
Ccdc191
c
ATRX
EZH2
H3K27me3
r=0.73
r=0.96
r=0.95
WT_R1
WT_R2
r=0.81
r=0.96
r=0.97
ΔRBR_R1
ΔRBR_R2
d
p=7.27e-57
Read Density EZH2 (Log2)
WT EZH2 peaks
ΔRBR Unique ATRX peaks
e
p<10e-100
Read Density H3K27me3 (Log2)
WT EZH2 peaks
ΔRBR Unique ATRX peaks

Supplementary Figure 5. Correlation between ATRX, EZH2 and H3K27 replicates in WT and ATRXΔRBR.

a) Genome browser view of *Fzd7* gene showing ATRX (purple), EZH2 (red), and H3K27me3 (teal) replicate CUT&RUN tracks from WT ATRX and ATRXΔRBR cells.

b) Genome browser view of *Qtrd1 and Ccdc191* genes showing ATRX (purple), EZH2 (red), and H3K27me3 (teal) replicate CUT&RUN tracks from WT ATRX and ATRXΔRBR cells. Peaks called for ATRX samples are shown (black bars).

c) Correlation scatterplot showing read densities for the union of peaks for CUT&RUN replicates as indicated on the axes (Log2 scale). Spearman correlation coefficient (r) is shown for each plot.

d) Boxplots of read densities (Log2 scale) for EZH2 enrichment at bona fide PRC2 target genes (n=29,042) and at genes that acquire ATRX peaks in ATRXΔRBR (n=4,512). Each boxplot represents median (center line), interquartile range (box), and min-max range (whiskers). P values are calculated using two-sided Student's t test.

e) Boxplots of read densities (Log2 scale) for H3K27me3 enrichment at bona fide PRC2 target genes (n=29,042) and at genes that acquire ATRX peaks in ATRXΔRBR (n=4,512). Each boxplot represents median (center line), interquartile range (box), and min-max range (whiskers). P values are calculated using two-sided Student's t test.

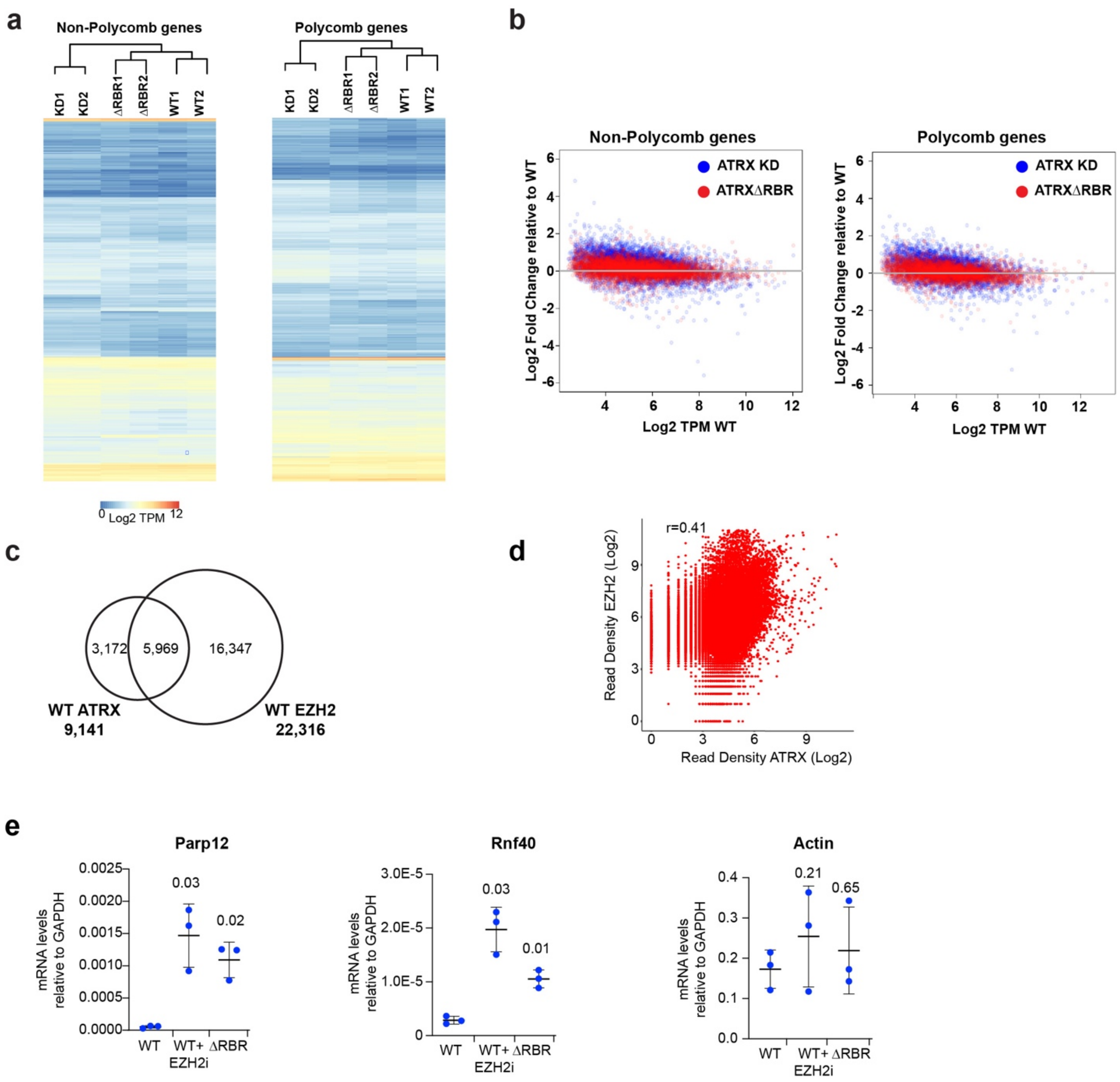

Supplementary Figure 6. Correlation between ATRX and EZH2 genome-wide and effect of ATRXΔRBR and ATRX KD on gene expression.

a) Heatmap showing expression of genes (Log2 TPM) classified as non-PRC2 and PRC2 targets based on presence of EZH2 in WT MEFs. Unsupervised clustering of samples indicated in columns.

b) TPM scatterplot of genes classified as non-PRC2 and PRC2 targets in WT MEFs. Log2 fold change in either ATRXΔRBR (red) or ATRXKD (blue) is plotted against average expression (Log2 TPM) in WT MEFs.

c) Peak overlap between ATRX and EZH2 in WT MEFs.

d) Correlation scatterplot showing read densities for the union of peaks for ATRX and EZH2 in WT MEFs as indicated on the axes (Log2 scale). Spearman correlation coefficient (r) is shown.
e) RT-PCR analysis of Parp12, Rnf40 and Actin in WT MEF before and after treatment with EZH2 inhibitor and in ATRXΔRBR. GAPDH was used for normalization. Data are presented as mean values +/- SEM. P values are calculated using two-sided Student's t test. . Source data are provided as a Source Data file.

Supplementary Table 1 – sequences of PCR primers used in this study.

Oligos	Sequence	Use
hATRX5625 Nco1 F	caccatggaaTTTCAGATGTTAAGTA	Pet101ATRX #2
hATRX6225 Xho1 R	gatctcgagCCCCTCACCTTTATAAATAAGG	Pet101ATRX #2
mATRX RBR1 deletion gRNA up F	CACCGTGTGTTGGATGATATCAAGA	pX459 ATRX up
mATRX RBR1 deletion gRNA up R	CTCTTGATATCATCCAACACACAAA	pX459 ATRX up
mATRX RBR1 deletion gRNA down F	CACCGAGCTAAGCAACCAGTGATTG	pX459 ATRX down
mATRX RBR1 deletion gRNA down R	CCAATCACTGGTTGCTTAGCTCAAA	pX459 ATRX down
MinSAT Fwd real time PCR	TTG GAA ACG GGA TTT GTA GA	RT-PCR
MinSAT Rev real time PCR	CGG TTT CCA ACA TAT GTG TTT T	RT-PCR
mGAPDH RT F	ATGAATACGGCTACAGCAACAGG	RT-PCR
mGAPDH RT R	CTCTTGCTCAGTGTCCTTGCTG	RT-PCR
CRISPR ATRX deletion fwd	gtcaaccagagcctctactgg	PCR
CRISPR ATRX deletion Rev	agccccaattctgctcatgg	PCR
Xist F	CAGAGTAGCGAGGACTTGAAGAG	RT-PCR
Xist R	GCTGGTTCGTCTATCTTGTGGG	RT-PCR
U1 snRNA F	GGAAATCATACTTACCTGGC	RT-PCR
U1 snRNA R	AAACGCAGTCCCCCACTACC	RT-PCR
Xist-F2	GGTTGATACTTGTGTGTGTATGGTGG	RT-PCR
Xist-R2	CGATGGGCTAAGGAGAAGAAA	RT-PCR
MBP-F-EcoRV	TGGATATCATGAAAATCGAAGAAGGTAAA	pCRII MBP
MBP-R-KpnI	GAGGTACCTGTTATAAATCAGCGATAACGC	pCRII MBP
MBP-F	TGGTGGCTACGCTCAATCTG	RT-PCR
MBP-R	AACAGCGATCGGGTAAGCAA	RT-PCR
Actin-RT-F	CTGTCCCTGTATGCCTCTG	RT-PCR
Actin-RT-R	ATGTCACGCACGATTTCC	RT-PCR
Parp12-RT-F	CAGAATGACCCTTCACTTTTGCCA	RT-PCR
Parp12-RT-R	GCTTGTGCCAAACTTGCACTCT	RT-PCR
Rnf40-RT-F	CTTTCAGGGAGGCCAGATTAC	RT-PCR
Rnf40-RT-R	CTCATTGTAGAGCAGGGAGAAC	RT-PCR

Supplementary Table 2 – Antibodies used in this study.

WB= Western Blot
IF= immunofluorescence
CLIP= crosslinking and immunoprecipitation
CUT&RUN= Cleavage Under Target and Release Using Nuclease

ANTIBODIES		
	SOURCE and catalog number	Usage
Rabbit-anti ATRX	This paper	WB 4ng/ml final concentration, IF 1:200, CUT&RUN 2μg
Mouse-anti HNRNPC	Santa Cruz Biotechnology sc-32308	WB 1:1000
Rabbit-anti LSD1	Abcam ab17721	WB 1:1000
Mouse-anti CBX5	Rauscher Lab (Wistar)	IF 1:200
Rabbit-anti NPM1	Sigma B0556	IF 1:500
Mouse-anti EZH2	BD Biosciences	WB 1:2000
Mouse-anti PML	Lieberman lab (Wistar)	IF 1:200
Rabbit-anti DAXX	Sigma D7810	WB 1:1000
Rabbit-anti-ATRX	Santa Cruz Biotechnology sc-15408	CLIP 2μg
Rabbit-anti Actin	Sigma A2066	WB 1:2000
Mouse-anti Tubulin	Sigma T9026	WB 1:1000
Rabbit-anti EZH2	Cell Signaling technology D2C9	CUT&RUN 2μg
Rabbit-anti H3K27me3	Cell Signaling technology C36B11	CUT&RUN 2μg

REFERENCES

1 Maenner, S. *et al.* 2-D structure of the A region of Xist RNA and its implication for PRC2 association. *PLoS Biol* **8**, e1000276, doi:10.1371/journal.pbio.1000276 (2010).
