# Supplementary material for: Disruption of ATRX-RNA interactions uncovers roles in ATRX localization and PRC2 function
Source: Nat Commun. 2020 May 6;11:2219. doi: 10.1038/s41467-020-15902-9 (PMC7203109; doi:10.1038/s41467-020-15902-9)
Supplement: Supplementary file 2 — Reporting Summary [file 41467_2020_15902_MOESM2_ESM.pdf]

nature research

Corresponding author(s): Kavitha Sarma

Last updated by author(s): Mar 6, 2020

Reporting Summary

Nature Research wishes to improve the reproducibility of the work that we publish. This form provides structure for consistency and transparency in reporting. For further information on Nature Research policies, see Authors & Referees and the Editorial Policy Checklist.

Statistics

For all statistical analyses, confirm that the following items are present in the figure legend, table legend, main text, or Methods section.

n/a	Confirmed	
☐	☒	The exact sample size (n) for each experimental group/condition, given as a discrete number and unit of measurement
☐	☒	A statement on whether measurements were taken from distinct samples or whether the same sample was measured repeatedly
☐	☒	The statistical test(s) used AND whether they are one- or two-sided<br>*Only common tests should be described solely by name; describe more complex techniques in the Methods section.*
☒	☐	A description of all covariates tested
☒	☐	A description of any assumptions or corrections, such as tests of normality and adjustment for multiple comparisons
☐	☒	A full description of the statistical parameters including central tendency (e.g. means) or other basic estimates (e.g. regression coefficient) AND variation (e.g. standard deviation) or associated estimates of uncertainty (e.g. confidence intervals)
☐	☒	For null hypothesis testing, the test statistic (e.g. F, t, r) with confidence intervals, effect sizes, degrees of freedom and P value noted<br>*Give P values as exact values whenever suitable.*
☒	☐	For Bayesian analysis, information on the choice of priors and Markov chain Monte Carlo settings
☒	☐	For hierarchical and complex designs, identification of the appropriate level for tests and full reporting of outcomes
☐	☒	Estimates of effect sizes (e.g. Cohen's d, Pearson's r), indicating how they were calculated

Our web collection on statistics for biologists contains articles on many of the points above.

Software and code

Policy information about availability of computer code

Data collection	No software was used
Data analysis	Bowtie2 version 2.2.9 : CUT&RUN Alignment<br>MaxQuant program version 1.6.10.43 : Mass Spectrometry<br>MACS2 version 2.1.1 : peak calling<br>ggplot2 version 3.2.1: graphs<br>STAR version 2.6.1 : RNA Seq alignment<br>pheatmap version 1.0.12: TPM heatmaps<br>RSEM version 1.3.1 : TPM calculation<br>ChAsE version 1.0.11 : Heatmaps<br>RStudio version 1.0.153: All basic data analysis<br>Deeptools version 3.2.1: metaplot<br>Bedtools version 2.26.0 : overlap analysis and read density calculations

For manuscripts utilizing custom algorithms or software that are central to the research but not yet described in published literature, software must be made available to editors/reviewers. We strongly encourage code deposition in a community repository (e.g. GitHub). See the Nature Research guidelines for submitting code & software for further information.

Data

Policy information about availability of data

All manuscripts must include a data availability statement. This statement should provide the following information, where applicable:
- Accession codes, unique identifiers, or web links for publicly available datasets
- A list of figures that have associated raw data
- A description of any restrictions on data availability

Sequencing data generated for this study have been deposited in the NCBI GEO as GSE130452

Field-specific reporting

Please select the one below that is the best fit for your research. If you are not sure, read the appropriate sections before making your selection.

☒ Life sciences ☐ Behavioural & social sciences ☐ Ecological, evolutionary & environmental sciences

For a reference copy of the document with all sections, see nature.com/documents/nr-reporting-summary-flat.pdf

Life sciences study design

All studies must disclose on these points even when the disclosure is negative.

Sample size	All sequencing experiments were performed with biological replicates as is the norm in the field. All fractionation and microscopy experiments were performed at different times using different batches of cells.
Data exclusions	No data was excluded from analysis
Replication	All genome wide experiments were reproduced twice and all PCR based experiments were reproduced at least 3 times (biological replicates). All other experiments microscopy, western blots were performed atleast 3 times using 3 independent biological samples.
Randomization	Not applicable
Blinding	not applicable

Reporting for specific materials, systems and methods

We require information from authors about some types of materials, experimental systems and methods used in many studies. Here, indicate whether each material, system or method listed is relevant to your study. If you are not sure if a list item applies to your research, read the appropriate section before selecting a response.

Materials & experimental systems

n/a	Involved in the study	
☐	☒	Antibodies
☐	☒	Eukaryotic cell lines
☒	☐	Palaeontology
☒	☐	Animals and other organisms
☒	☐	Human research participants
☒	☐	Clinical data

Methods

n/a	Involved in the study	
☐	☒	ChIP-seq
☒	☐	Flow cytometry
☒	☐	MRI-based neuroimaging

Antibodies

Antibodies used	All antibodies used in the study are listed with amounts/dilutions used and application in Supplementary Table 2.
Validation	All commercially available antibodies were already validated and used according to the manufacturer instructions. ATRX antibody was extensively characterized and validated in the present study.

Eukaryotic cell lines

Policy information about cell lines

Cell line source(s)	MEFs and 293 cells used in this study were obtained from Jeannie Lee's laboratory (MGH) and have been described previously in Sarma et al, Cell 2014.

Authentication	Knockdown cell lines were assessed by western blot. ATRX RBR deletion cell lines were authenticated with restriction analysis, sanger sequencing and western blotting.
Mycoplasma contamination	Cell lines tested negative for mycoplasma contamination at the beginning of the study
Commonly misidentified lines (See ICLAC register)	No cell lines used are listed in the database of commonly misidentified cell lines

ChIP-seq

Data deposition

☒ Confirm that both raw and final processed data have been deposited in a public database such as GEO.

☒ Confirm that you have deposited or provided access to graph files (e.g. BED files) for the called peaks.

Data access links *May remain private before publication.*	https://www.ncbi.nlm.nih.gov/geo/query/acc.cgi?acc=GSE130452
Files in database submission	All the raw and processed data files can be accessed with the link above. This data is now publicly available.
Genome browser session (e.g. UCSC)	No longer applicable

Methodology

Replicates	Biological replicates were sequenced for each sample.
Sequencing depth	Approximately 15-25 million reads were obtained for each sample. Samples are paired end 75bp.
Antibodies	All antibody information is available in supplementary table 2
Peak calling parameters	Peaks were called for each sample using MACS2 using the parameters: --broad --broad-cutoff 0.1
Data quality	Peaks were considered with p<0.1
Software	reads were aligned to the mouse reference genome mm10 using Bowtie2 version 2.2.9 with default parameters.

October 2018
